# Non-fluorinated phosphonium-sulfonate ionic liquids as candidates for thermally demanding applications: thermal stability, viscosity and liquid-state temperature ranges

**DOI:** 10.1039/d6ra08417f

**Published:** 2026-11-04

**Authors:** A. Centeno-Pedrazo, J. Perez-Arce, B. Iliev, T. J. S. Schubert, S. Lorenz, A. Agrelo-Lestón, M. Haumann, M. Distaso, C. Len, I. Zabala, S. Doppiu, E. J. Garcia-Suarez

**Affiliations:** a Center for Cooperative Research on Alternative Energies (CIC EnergiGUNE), Basque Research and Technology Alliance (BRTA) Álava Technology Park, Albert Einstein 48 01510 Vitoria-Gasteiz Spain esuarez@cicenergigune.com jperez@cicenergigune.com; b IOLITEC Ionic Liquids Technologies GmbH Salzstrasse 184 74076 Heilbronn Germany; c Friedrich-Alexander-Universität Erlangen-Nürnberg (FAU), Lehrstuhl für Chemische Reaktionstechnik (CRT) Egerlandstr. 3 91058 Erlangen Germany; d Helmholtz-Institut Erlangen-Nürnberg for Renewable Energy (IET-2), Forschungszentrum Jülich GmbH Cauerstraße 1 Erlangen 91058 Germany; e Chimie ParisTech, PSL Research University, CNRS, Institute of Chemistry for Life and Health Sciences 11 Rue Pierre et Marie Curie F-75005 Paris France; f SENER Ingeniería y Sistemas S.A. Zugatzarte Etorb. 56 48930 Getxo Spain; g IKERBASQUE, Basque Foundation for Science Plaza Euskadi 5 48009 Bilbao Spain

## Abstract

Room-temperature ionic liquids with high thermal stability are attractive candidates for applications requiring operation at elevated temperatures. In this work, a series of non-fluorinated phosphonium-sulfonate ionic liquids was synthesized and systematically characterized to establish structure–property relationships and evaluate their potential as thermally robust fluids. Thermal stability, density, and viscosity were evaluated to determine the influence of cation and anion structure. Under dynamic conditions, all ionic liquids showed encouraging thermal stability, with *T*_5%_ values of up to 406 °C. Isothermal measurements revealed that four of the seven studied ionic liquids exhibited weight losses equal to 2.6 wt% after 12 h at 300 °C. It has been found that the cation and anion strongly influence thermal stability; in the tributyltetradecylphosphonium ([(C_4_)_3_C_14_P]) cation-based ionic liquids, the presence of an aromatic ring in the anion enhanced stability, highlighting the importance of anion structure. In dodecylbenzenesulfonate ([DBS])-based ionic liquids, the cation bearing the longest alkyl chains exhibited the lowest weight loss under isothermal conditions. Density and viscosity were also strongly dependent on ionic liquid structure and temperature. Longer alkyl chains in the cation led to lower densities, whereas the same trend was observed for the anion within the aromatic subseries. Viscosity decreased strongly with temperature and was mainly controlled by the anion, with [DBS]-based ionic liquids exhibiting the highest values. Within the aromatic-anion subseries, viscosity increased with alkyl chain length, whereas the cation effect was less systematic. Overall, the integration of melting behaviour, isothermal thermal stability, density, viscosity, and viscosity-temperature modelling establishes a structure-guided thermophysical dataset for non-fluorinated phosphonium-sulfonate ionic liquids and defines temperature ranges over which the ILs remain liquid and thermally stable, extending up to 300 °C. For [(C_4_)_3_C_14_P][DBS], an empirical stability model based on isothermal data predicted 1 wt% mass loss after 10 h at 279 °C, providing a quantitative relationship between exposure temperature, time, and degradation. Together, these results highlight the potential of these ILs for applications requiring thermally robust liquids.

## Abstract 2

Room-temperature ionic liquids with high thermal stability are attractive candidates for applications requiring operation at elevated temperatures.

## Graphical abstract

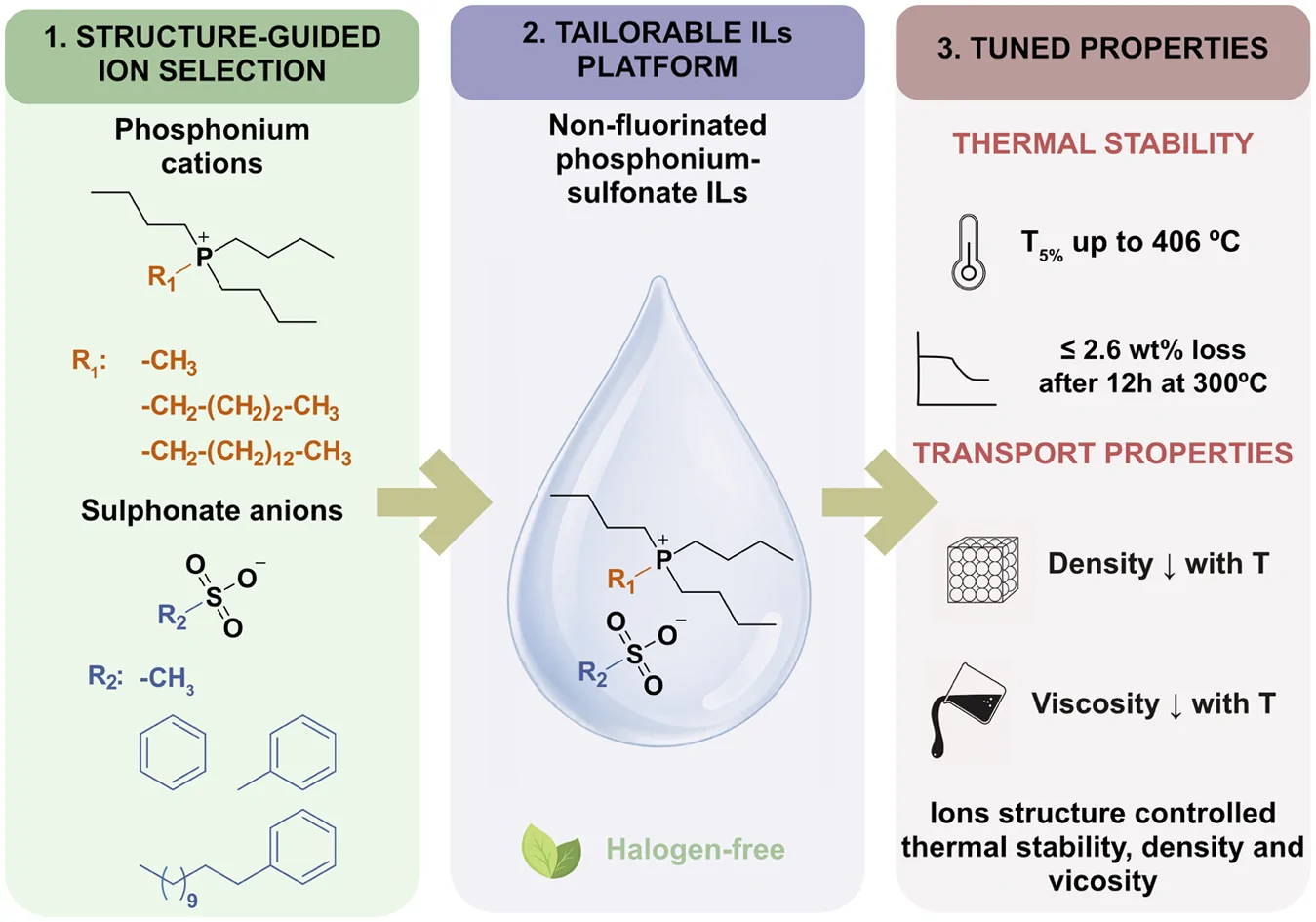

## 1. Introduction

As modern technologies advance toward greater efficiency and increasingly demanding operating conditions, the development of fluids capable of operating at elevated temperatures has become a major challenge in fields such as power generation, thermal management, and high-temperature chemical processing. For such applications, thermal stability alone is not sufficient. Candidate fluids must combine resistance to degradation under prolonged thermal exposure with liquid-state behavior over the relevant temperature range and viscosity values compatible with fluid handling. In this context, ionic liquids (ILs) have emerged as promising candidates due to their highly tunable nature. Therefore, the identification of structure–property relationships that simultaneously account for thermal stability, density, and viscosity is essential for the rational selection of ILs as candidate fluids for elevated-temperature applications. This growing interest reflects the potential of ILs as advanced functional fluids for demanding energy applications, as highlighted in the scientific literature.^1–4^

ILs are commonly arbitrarily defined as salts that are liquid at temperatures below 100 °C. They exhibit a combination of promising physicochemical properties for thermal applications, including negligible vapour pressure, low-melting points, reduced flammability, and tunability through variation of their cation–anion combinations. However, the potential of ILs is not limited to these properties. Along with these advantages, numerous studies have reported that they exhibit a very high degree of chemical and thermal stability, which allows their use as high-temperature fluids.^5^ In this context, phosphonium-based ILs have attracted particular attention due to their superior thermal stability.^6,7^ Additionally, many high-valent organophosphorus compounds act as effective flame retardants, highlighting the potential of phosphonium-based ILs as high-performance materials for thermally demanding environments.^8^ However, despite this promising stability, it has been recognized that many ILs can undergo degradation when exposed to temperatures lower than critical or onset temperatures for extended periods of time.^9^

To evaluate the thermal stability of ILs, thermogravimetric analysis (TGA) under dynamic conditions is typically employed, where mass loss is measured as the temperature increases at a constant heating rate (usually 5–20 °C min^−1^).^10^ It is well known that the atmosphere, mass and/or heating rate used in TGA experiments can significantly affect the observed mass loss and, therefore, the evaluation of thermal stability. Consequently, TGA data obtained under different experimental conditions should be compared with caution, as variations in the experimental process can influence the measured decomposition temperatures. This issue becomes even more complicated by inconsistencies in how thermal stability is reported in the literature, with some studies using onset temperatures, others reporting temperatures at 1–5 wt% loss, and others relying on different weight loss-temperature criteria.^11^ Moreover, dynamic analyses may not be the most appropriate methodology for evaluating thermal stability, because the sample is exposed to each temperature only for a short period, typically from seconds to a few minutes. Thus, the decomposition temperatures obtained from these measurements often overestimate the real thermal stability of ILs. In fact, numerous studies have shown that isothermal stability experiments can differ significantly from that suggested by dynamic experiments.^9,12,13^ Therefore, in practical applications, significantly longer exposure times must be considered to obtain a more representative evaluation of thermal stability.

Among thermally robust ILs, some of the most remarkable examples are those developed by Davis's group.^14–18^ These peraryl onium cation-based (tetraarylphosphonium, triphenylsulfonium, and triphenyl(*P*,*P*,*P*-triphenylphosphine imidato-*κN*)-phosphorus) mesothermal ILs demonstrated exceptional long-term thermal stability, remaining stable up to 90 days at 300 °C. However, all the reported salts are solids at room temperature, only a few become liquid near 100 °C, and the majority require even higher temperatures to reach the liquid state, thereby restricting their operating temperature window. In this context, Musozoda and co-workers reported π-conjugated triphenylphosphonium ILs, designed to balance low melting points with high thermal stability.^19^ Although this strategy developed ILs with a wide liquid range, many of the resulting compounds still exhibited melting points above 100 °C, highlighting the challenge of combining high thermal stability with low melting points (R.T. or lower) thereby limiting their applicability window. This further illustrates the significant challenge of designing ILs that combine very high thermal stability with sufficiently low melting points to remain liquid at or near room temperature.

With the increasing interest in thermal fluids, designing ILs that combine superior thermal stability with low melting points remains a significant challenge. In ILs, the melting point can be reduced by increasing the structural asymmetry and conformational flexibility of the ions, thereby disrupting efficient crystal packing.^20,21^ In this context, the incorporation of long aliphatic chains is known to promote the formation of room-temperature ILs. In contrast, aromatic structures are often associated with enhanced thermal stability, but their greater rigidity can also result in higher melting points, which may limit room-temperature liquidity and narrow the liquid operating window.^5^ For this reason, aliphatic phosphonium cations were selected in this work to favor the formation of ILs with low melting points (R.T. or lower). In this sense, the nature of the anion plays a key role. Several anions can be combined with highly stable phosphonium cations, including halides (F^−^, Cl^−^, Br^−^, I^−^), tetrafluoroborate (BF_4_^−^), hexafluorophosphate (PF_6_^−^), trifluoromethanesulfonimide (TFSI), and sulfonate (RSO_3_^−^).

Among these, sulfonate anions offer several advantages from both molecular design and sustainability perspectives. Unlike widely used fluorinated anions such as, [TFSI], [BF_4_] or [PF_6_], sulfonate anions can provide a non-fluorinated platform for the development of ILs with tunable physicochemical properties. Moreover, the development of non-fluorinated alternatives is attractive from a sustainability perspective, since some commonly used fluorinated anions present substantial environmental challenges, as they can lead to the release of toxic and persistent pollutants.^22^ In addition, the sulfonate framework can be readily modified with aromatic or alkyl substituents, providing considerable flexibility for adjusting thermal stability, density, and viscosity.^23,24^ Taken together, these features make phosphonium-sulfonate ILs a promising family for the development of thermally stable ILs with broad liquid-phase temperature range. Many of these ILs remain liquid at or near room temperature, which is particularly advantageous for applications requiring fluids that can operate over wide temperature ranges. Although phosphonium-sulfonate ILs have been previously reported, systematic studies addressing their thermal stability under isothermal conditions and physicochemical properties remain scarce.^25,26^

In this work, a series of non-fluorinated phosphonium-sulfonate ILs was synthesized and systematically investigated to establish structure–property relationships relevant to the selection of candidate fluids for elevated-temperature applications. The study integrates dynamic and isothermal thermal stability, density, viscosity, and viscosity-temperature modelling to determine how cation–anion structure controls the liquid-phase operating range. Particular attention was given to isothermal stability at 300 °C for 12 h and to the temperature dependence of viscosity, since these parameters are critical for assessing thermally robust fluids under application-relevant conditions. By combining these criteria, this work provides a structure-guided framework for selecting non-fluorinated phosphonium-sulfonate ILs as candidate fluids for thermally demanding applications.

## 2. Experimental

### 2.1. Chemicals and materials

Tributylmethylphosphonium chloride ([(C_4_)_3_C_1_P]Cl), tetrabutylphosphonium chloride ([(C_4_)_4_P]Cl), tributyltetradecylphosphonium chloride ([(C_4_)_3_C_14_P]Cl), and trihexyltetradecylphosphonium chloride [(C_6_)_3_C_14_P]Cl were supplied by IOLITEC with a purity higher than 95%. Methanesulfonic acid (MS) (≥99%), *p*-toluenesulfonic acid (Ts) (97%), benzenesulfonic acid (BS) (≥97%), and sodium dodecylbenzenesulfonate (DBS) (technical grade) were obtained from Sigma-Aldrich. The commercial sodium alkylbenzene sulfonates used as starting material consist of a mixture of alkylbenzene sulfonate isomers.

### 2.2. Synthesis of phosphonium-sulfonate ionic liquids

Since most of the ILs are poorly water soluble, they were synthesized by aqueous anion exchange, followed by an extraction with dichloromethane according to the following general procedure (for details see Fig. S1 in SI). To a solution of phosphonium chloride precursors [(C_4_)_3_C_1_P]Cl; [(C_4_)_4_P]Cl; ([(C_4_)_3_C_14_P]Cl; [(C_6_)_3_C_14_P]Cl), (0.1 mol) in 1 L of dichloromethane was added 0.11 mol of the corresponding sodium salt of the anion acid (methanesulfonic acid, dodecylbenzenesulfonic acid, benzenesulfonic acid, *p*-methylbenzenesulfonic acid) in 100 mL of water. The biphasic mixture was stirred for 24 hours, then separated in a funnel, and the organic phase was washed 4 times with 50 mL of deionized water. The organic phase was separated, evaporated under reduced pressure, and dried under vacuum at 40 °C.

### 2.3. NMR spectroscopy

^1^H, ^13^C{^1^H} and ^31^P{^1^H} NMR spectra were recorded on a BRUKER Avance II 300 MHz (^1^H at 300 MHz and ^13^C{^1^H} at 75 MHz). The sample (15–20 mg) was placed in a 5 mm NMR tube with 0.6 mL of dichloromethane-*d*_2_ as deuterated solvent. The chemical shifts were reported in ppm (*δ*) referenced to the chemical shifts of residual solvent resonances (^1^H and ^13^C) or 85% H_3_PO_4_ (^31^P{^1^H}), respectively.

### 2.4. Melting point measurements

Melting points were measured by differential scanning calorimetry (DSC) analysis carried out using DSC Q2500 from TA Instruments with sealed Al crucibles. DSC scans consisted of heating the sample to 40 °C, followed by a cooling ramp from 40 °C to −80 °C and a subsequent heating ramp from −80 °C to 40 °C, both at 5 °C min^−1^.

### 2.5. Thermogravimetric analysis

Thermal stability was evaluated by simultaneous thermal gravimetric analysis (STA) using Netzsch STA 449 F3 Jupiter under nitrogen flow (60 mL min^−1^). In a typical dynamic analysis, the sample (40–60 mg) was heated from 25 °C up to 120 °C with a heating rate of 10 °C min^−1^, and kept under isothermal conditions (120 °C, 1 h) to eliminate moisture and/or residual solvent, if any. Then, the sample was heated from 120 °C to 600 °C at a rate of 10 °C min^−1^. Decomposition temperature was determined by the onset temperature defined according to ISO 11358-1 as “the point of intersection of the starting-mass baseline and the tangent to the TGA curve at the point of maximum gradient”.^27^ Isothermal measurements were performed at 300 °C by heating the sample from room temperature to 120 °C with a heating rate of 10 °C min^−1^ and kept under isothermal conditions (120 °C, 1 h) to eliminate moisture and/or impurities. Then, the sample was heated from 120 °C to 250 °C with a heating rate of 20 °C min^−1^. Finally, the sample was heated from 250 °C to 300 °C at a rate of 5 °C min^−1^ and kept at this temperature for 12 h. For [(C_4_)_4_P][DBS] TG209F1 Libra Thermogravimetric Analyzer from NETZSCH was employed under the same conditions. The experimental uncertainty of the thermogravimetric analysis was estimated to be approximately 3%.

### 2.6. Density measurements

The experimental densities of those ILs that are liquid at room temperature were determined by using an Anton Paar DMA 4200 M densimeter. The temperature range was from 25 to 150 °C at atmospheric pressure, and measurements were acquired in duplicate.

### 2.7. Viscosity measurements

Viscosity measurements were performed using a TA Instruments Discovery HR-1 rheometer equipped with a 25 mm parallel-plate geometry over the temperature range of 25–200 °C. For each IL and at each temperature, a shear-rate sweep was first performed to determine the flow behaviour and identify the Newtonian region. Once the corresponding shear-rate range was established, an appropriate shear rate within this region was selected, and the viscosity was measured under these conditions to ensure that the reported values corresponded to the Newtonian flow regime.

### 2.8. Water content determination

The water content of the ILs was determined by coulometric Karl-Fischer titration using a C20 coulometric KF titrator from Mettler Toledo. Approximately 0.1 g of each IL was accurately weighed and dissolved in 1 mL of anhydrous dichloromethane. The resulting solution was quantitatively introduced into the titration vessel, and the water content was determined coulometrically until automatic endpoint detection. The water contribution from dichloromethane was determined from three independent blank measurements, and the average value was subtracted from the total water measured for the IL solution. The final water content was calculated relative to the mass of IL used. Three independent determinations were performed for each IL at room temperature.

### 2.9. Chloride content determination

The chloride content in the IL samples was quantified using a Combustion Ion Chromatography (CIC) system. The setup consisted of an Analytik Jena combustion module coupled with a Metrohm 920 absorption module and a Metrohm 930 Compact IC Flex ion chromatograph equipped with chemical suppression and a conductivity detector. In a typical procedure, approximately 25 mg of the sample was weighed into a quartz boat. The sample was combusted at 1050 °C under an oxygen flow (300 mL min^−1^) and an argon flow (100 mL min^−1^). The evolved gases were trapped and absorbed in an aqueous hydrogen peroxide solution (100 ppm). Then, an aliquot of 200 µL from the absorption solution was injected into the ion chromatograph. Anionic separation was achieved using a Metrohm Metrosep A Supp 7–150 anion-exchange column operating at 45 °C. Isocratic elution was carried out using an aqueous solution of sodium carbonate (3.6 mmol L^−1^) as the mobile phase at a constant flow rate (0.8 mL min^−1^). Quadruplicate measurements were performed for each sample.

### 2.10. CHNS elemental analysis

Elemental analyses were performed using a Thermo Scientific FLASH 2000 Elemental Analyzer in CHNS configuration for the simultaneous determination of carbon, hydrogen, nitrogen, and sulfur. The samples were combusted at 950 °C, and the resulting combustion products were separated chromatographically and detected using a thermal conductivity detector (TCD). Duplicated measurements were performed for each sample. The P and O content was determined as the difference between the % C, H, S and 100%.

## 3. Results and discussion

### 3.1. Synthesis and characterization of ionic liquids

Seven phosphonium-sulfonate ILs (Fig. 1) were synthesized. The ILs were designed to combine chemical robustness with structural flexibility, allowing the tuning of their physicochemical properties. For this purpose, phosphonium cations with different alkyl chain lengths, such as tributylmethylphosphonium and tributyltetradecylphosphonium, were selected together with sulfonate anions containing aliphatic alkyl chains of different lengths, with or without aromatic rings.

**Fig. 1:**
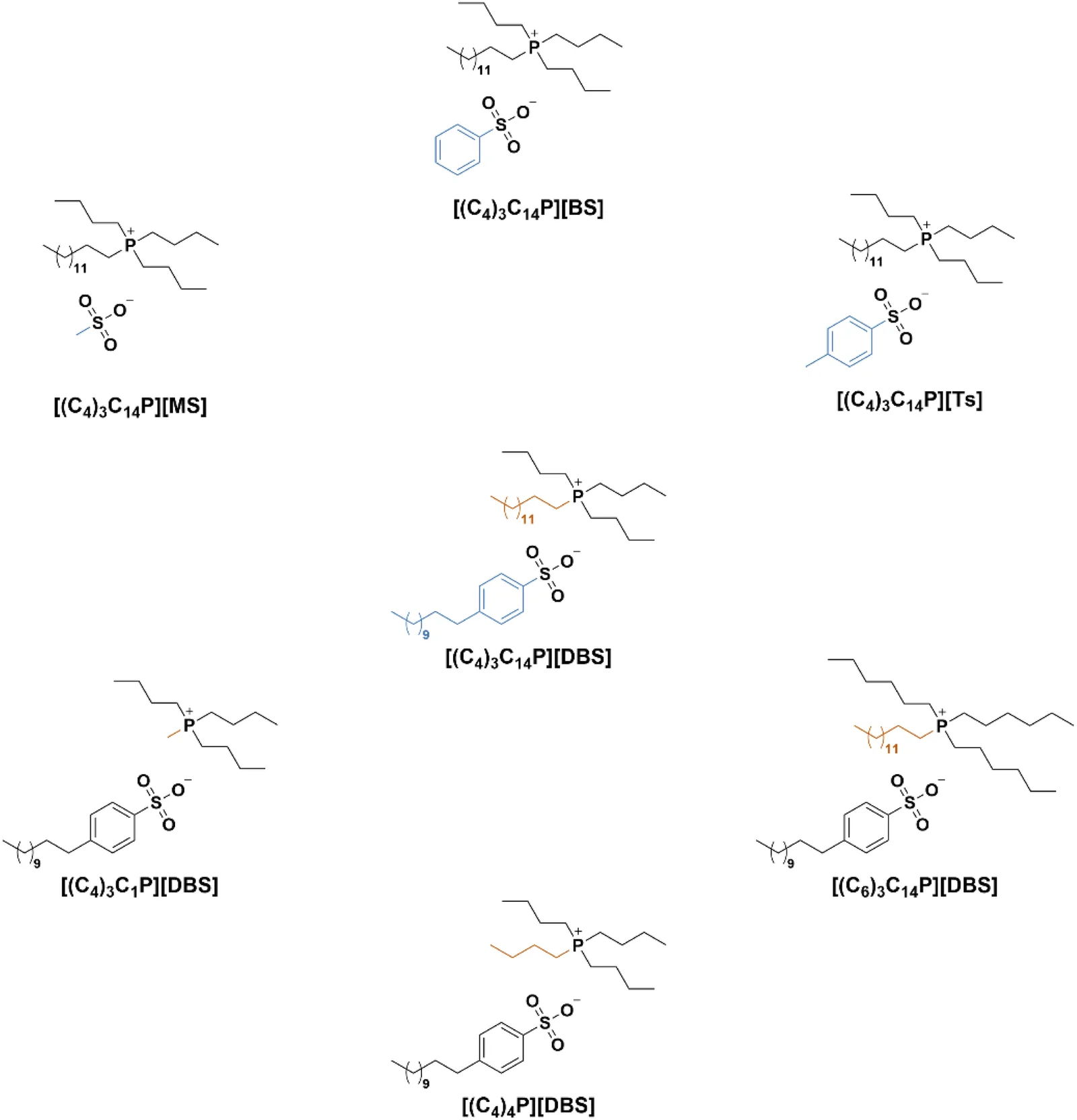
Prepared phosphonium-sulfonate ionic liquids employed in this work.

All ILs were prepared from commercially available starting materials through a one-step anion-exchange procedure and purified by aqueous washing and solvent removal (Fig. S1). The chemical structure of the prepared ILs was confirmed by ^1^H, ^13^C{^1^H} and ^31^P{^1^H} NMR spectroscopy (Fig. S2), in addition, the purity of the ILs was supported by elemental analysis (Table S4).

### 3.2. Thermal stability behaviour of studied ILs

#### 3.2.1. Stability of studied ILs under dynamic conditions

The short-term thermal stability of the phosphonium-sulfonate ILs was determined by dynamic STA analysis. Decomposition onset temperature (*T*_onset_), the temperature at 5% weight loss (*T*_5%_) and temperature at maximum decomposition rate (*T*_peak_) are shown in Table 1 and depicted in Fig. S3. Since *T*_onset_ may overestimate practical thermal stability, and *T*_peak_ occurs only after significant decomposition has taken place, *T*_5%_ was used as a more reliable parameter for evaluating the short-term thermal stability of ILs. All phosphonium-sulfonate ILs exhibited high thermal stability, with a temperature for 5% weight loss ranging from 385 °C to 406 °C (Fig. S3). Among the [(C_4_)_3_C_14_P]-based ILs, systems containing an aromatic functionality in the anion showed higher thermal stability than those without an aromatic structure (Table 1 entries 1 *vs.* 2–4). This behaviour may be tentatively associated with two factors reported in the literature: improved charge delocalization, which can reduce anion nucleophilicity, and increased steric hindrance linked to aromatic structures.^28,29^ Accordingly, Blanco *et al.* reported that the higher degree of charge delocalization in aromatic anions decreases their nucleophilicity and consequently their ability to promote cation decomposition,^28^ whereas Huang *et al.* stated that higher steric hindrance around the phosphonium centre lowers the interactions between anion–cation thus limiting decomposition processes.^29^ Hence, the higher stability observed for the aromatic systems in our study may result from the combined effect of lower anion nucleophilicity and increased steric hindrance, both of which reduce, based on precedent scientific literature, the probability of resulting in ILs decomposition. It is worth noting that the differences in *T*_5%_ among the ILs bearing aromatic anions were minimal (Table 1 entry 2 *vs.* entry 3 *vs.* entry 4). The observed slight decrease in thermal stability with increasing carbon chain may fall within the estimated experimental uncertainty that is around 3% and is therefore not considered significant. For [DBS]-based ILs, the influence of alkyl chain length on *T*_5%_ was not significant. Therefore, no clear correlation between chain length and thermal stability can be established, as the differences in decomposition temperature values were below 15 °C. This observation suggests that the initial degradation step is associated with the anion rather than the cation. Nevertheless, as stated before, it is important to emphasize that the thermal stability trend depends strongly on the parameter selected for comparison (*e.g.*, *T*_onset_, *T*_5%_, *T*_peak_). In fact, as observed in the [(C_4_)_3_C_14_P] and [DBS] families (Table 1), the relative thermal stability rank varies considerably when shifting from one measurement temperature to another, hindering direct comparisons of thermal stability. Therefore, the stability of ILs and, more specifically, of the phosphonium-sulfonate ILs investigated in this study, would be more appropriately evaluated by means of isothermal studies.

**Table 1:** Melting point and thermal stability parameters of the investigated ionic liquids

Entry	Cation	Anion	Physical state^a^	*T*_m_ b (°C)	*T*_onset_ c (°C)	*T*_5%_ d (°C)	*T*_peak_ e (°C)	wt. loss^f^ (%)
1	[(C_4_)_3_C_14_P]	[MS]	L	<−50^g^	405	387	427	7.3
2		[BS]	L	<−50^g^	418	406	442	2.4
3		[TS]	L	<−50^g^	409	399	430	1.6
4		[DBS]	L	<−50^g^	408	397	436	2.1
5	[(C_4_)_3_C_1_P]	[DBS]	L	<−50^g^	423	385	446	5.3
6	[(C_4_)_4_P]		L	<−50^g^	414	396	437	6.5
7	[(C_6_)_3_C_14_P]		L	<−50^g^	409	395	429	2.6

a Physical state: liquid (L).

b *T*_m_: melting point temperature.

c *T*_onset_: onset decomposition temperature.

d *T*_5%_: temperature at 5% weight loss.

e *T*_peak_: temperature at maximum decomposition rate.

f wt. loss: weight loss after 12 h at 300 °C.

g *T*_m_ < −50 °C indicates that no melting peak was detected above this temperature under the DSC conditions employed.

#### 3.2.2. Stability of studied ILs under isothermal conditions at 300 °C for 12 h

The isothermal stability of the phosphonium-sulfonate ILs was evaluated at 300 °C for 12 h using STA equipment. The stability was assessed from the weight loss recorded during this final isothermal stage, whereas the weight loss observed during the preceding treatment at 120 °C for 1 h was excluded, as it may be associated with residual moisture and/or solvent. The weight loss measured during the isothermal test is shown in Fig. 2. As can be observed, all the ILs lost weight at temperatures notably lower than *T*_5%_ (Table 1). This observation highlights the need to interpret dynamic analysis results with caution and highlights the importance of isothermal experiments to reliably assess the thermal stability of ILs at a given working temperature for the required working time.

**Fig. 2:**
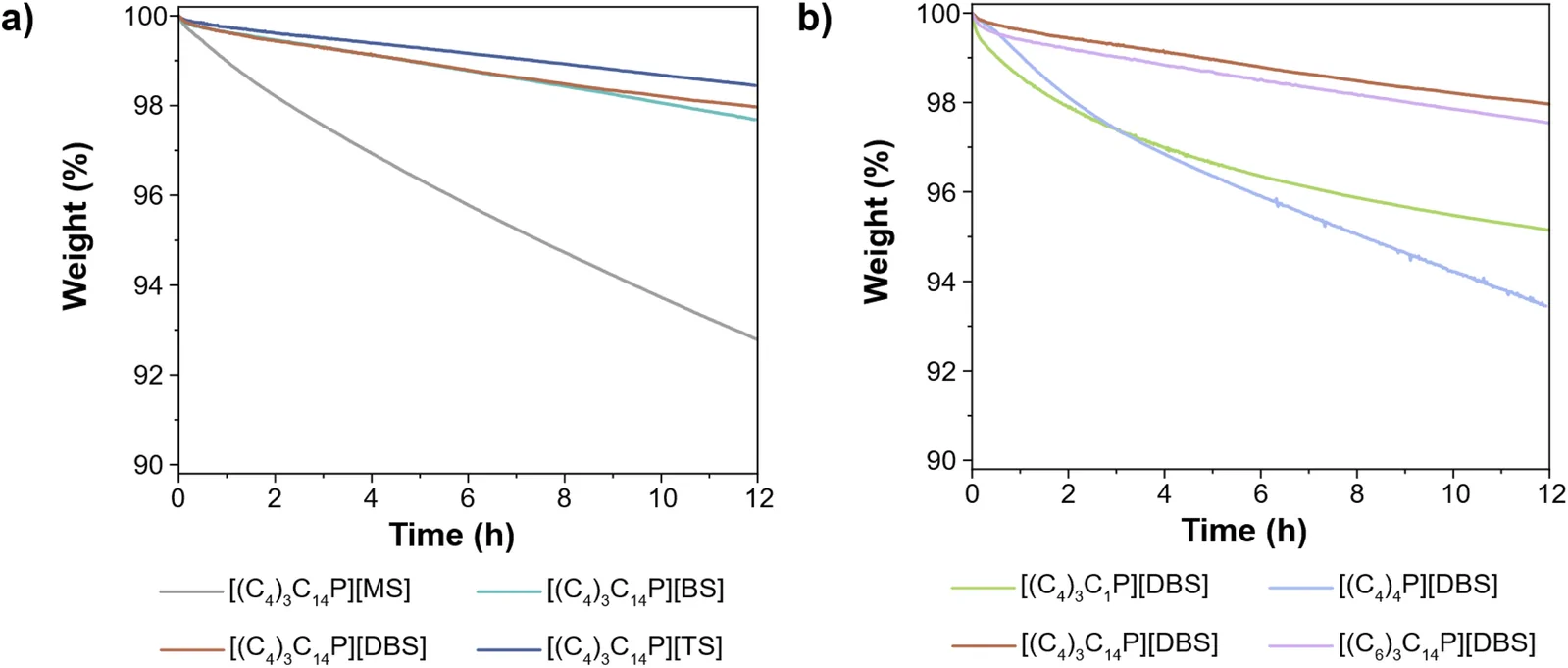
[(C_4_)_3_C_14_P]-based ILs (a) and [DBS]-based ILs (b) isothermal thermogravimetric analysis at 300 °C.

For [(C_4_)_3_C_14_P]-based ILs, the weight loss after 12 h at 300 °C ranged from 1.6% to 7.3%, corresponding to a mass loss below 1% h^−1^ in all cases (Fig. 2a). A similar trend to that observed under dynamic conditions was identified, with the presence of an aromatic ring in the anion increasing thermal stability. Among the ILs containing aromatic anions (Table 1 entry 2–4), the differences in weight loss did not exceed 0.6%, indicating very similar stability within this group. These results highlight the importance of anion delocalization and suggest that it is a key factor in the design of thermally stable ILs as already reported in literature.^28^ For the [DBS]-based ILs, a comparable trend to that observed under dynamic conditions was found, with cations bearing longer aliphatic chains showing the lowest weight loss under isothermal conditions (Fig. 2b and Table 1 entry 4–7). The observed trend in thermal stability under isothermal conditions follows the order: [(C_4_)_3_C_14_P][TS] > [(C_4_)_3_C_14_P][DBS] > [(C_4_)_3_C_14_P][BS] > [(C_6_)_3_C_14_P][DBS] > [(C_4_)_3_C_1_P][DBS] > [(C_4_)_4_P][DBS] > [(C_4_)_3_C_14_P][MS]. Overall, the isothermal thermal analysis indicates that selected phosphonium-sulfonate ILs are promising candidates for further evaluation in high-temperature applications, as four systems ([(C_4_)_3_C_14_P][TS], [(C_4_)_3_C_14_P][BS], [(C_4_)_3_C_14_P][DBS] and [(C_6_)_3_C_14_P][DBS]) exhibited very high stability, with weight losses ≤2.6% after 12 h at 300 °C (≈0.21% h^−1^). However, it should be noted that the 12 h isothermal experiment provides a comparative screening of thermal stability rather than a full lifetime assessment under application-specific operating conditions. To validate the thermal analysis, [(C_4_)_3_C_14_P][DBS] and [(C_4_)_4_P][DBS] were analyzed by ^1^H and ^31^P NMR before and after the 12 h isothermal treatment at 300 °C (Fig. S5). The ^1^H NMR spectra remained essentially unchanged after the thermal treatment for [(C_4_)_3_C_14_P][DBS] and [[(C_4_)_4_P][DBS]. However, for [(C_4_)_3_C_14_P][DBS], a new low-intensity peak was observed around 2.0 ppm after 12 h at 300 °C, indicating that a minor chemical modification cannot be excluded. Nevertheless, for both ILs, no significant changes were observed in the ^31^P{^1^H} NMR spectra. Overall, these results indicate that the main IL structures were largely preserved after 12 h at 300 °C under the experimental conditions employed. Additionally, residual chloride contents were determined by combustion ion chromatography to evaluate their possible influence on thermal stability (Table S1), since chloride may promote dealkylation of alkylphosphonium cations at elevated temperatures. No direct correlation was observed between residual chloride content and thermal stability. In both the [(C_4_)_3_C_14_P]- and [DBS]-based series, ILs with higher chloride contents were among the most thermally stable systems. This indicates that residual chloride is not the main factor controlling the stability trends, which appear to be more strongly governed by the cation–anion structure.

#### 3.2.3. Density

Density (*ρ*) is a key thermophysical property because it depends on cation–anion interactions and molecular packing. Its determination provides valuable insight into structure–property relationships and is also essential for the design of processes involving these fluids.^30^ The density of ILs is strongly affected by both temperature and ion structure, reflecting differences in packing efficiency, molecular geometry, and intermolecular interactions.^31^ For room-temperature ILs, densities were measured at three temperatures between 25 °C and 150 °C, and the respective values are depicted in Fig. 3 and detailed in Table S2.

**Fig. 3:**
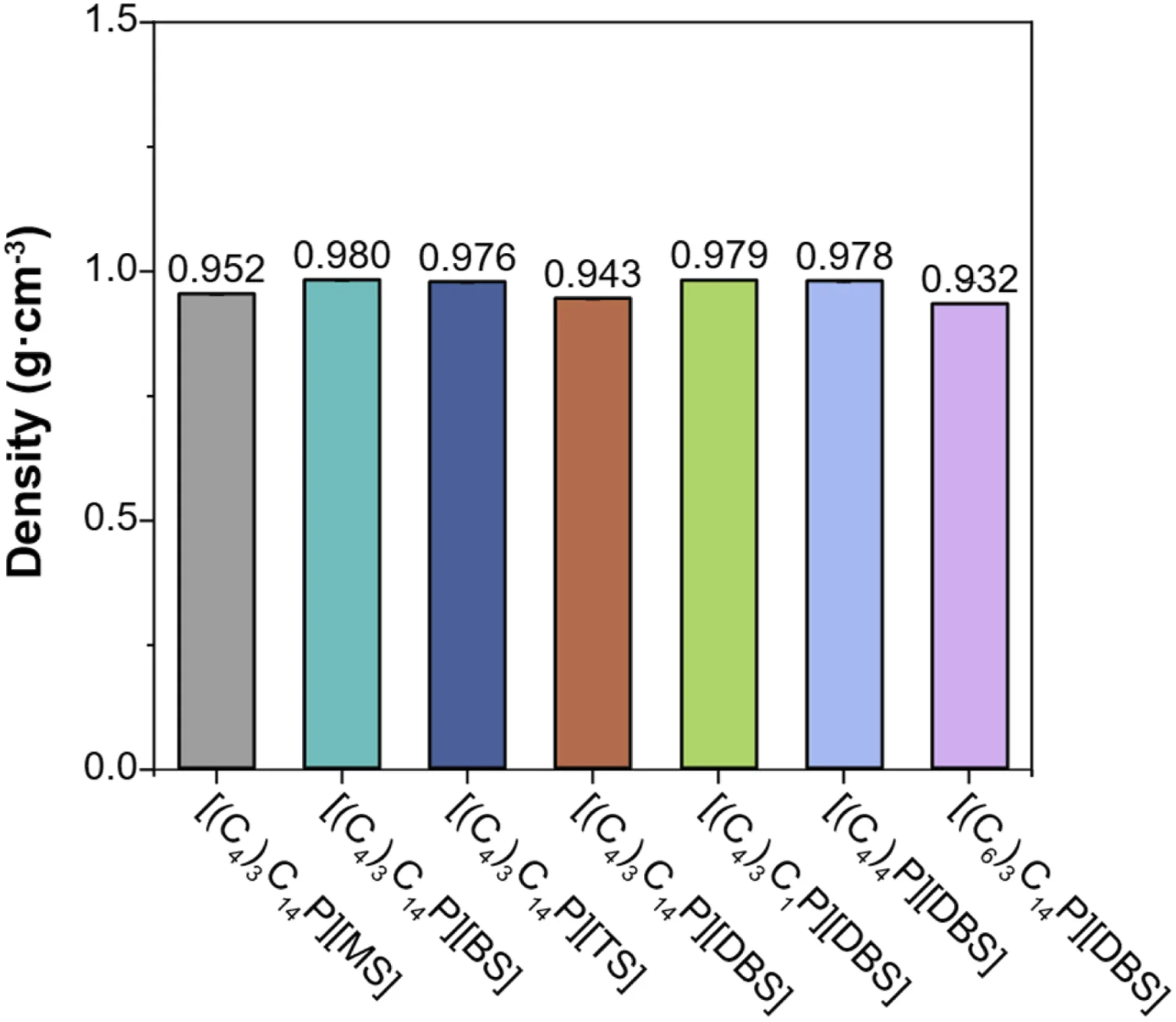
Density at 25 °C of phosphonium-sulfonated ionic liquids.

Variations in cation and anion structure resulted in a density range, from 0.932 to 0.980 g cm^−3^ (Fig. 3). The observed trend in density follows the order: [(C_4_)_3_C_14_P][BS] ≈ [(C_4_)_3_C_1_P][DBS] ≈ [(C_4_)_4_P][DBS] ≈ [(C_4_)_3_C_14_P][TS] > [(C_4_)_3_C_14_P][MS] > [(C_4_)_3_C_14_P][DBS] > [(C_6_)_3_C_14_P][DBS]. Phosphonium-sulfonate ILs with longer aliphatic cation chains exhibited lower densities. A similar trend was observed for the anion, but only within the aromatic-anion subseries, where increasing alkyl chain length also led to lower density.

The influence of temperature on the density of the phosphonium-sulfonate ILs is shown in Fig. 4. As commonly reported for ILs, the density of all studied ILs decreased linearly with increasing temperature. This behaviour is linked to the thermal expansion due to weaker intermolecular interactions at higher temperatures, which lead to less efficient molecular packing.^32^

**Fig. 4:**
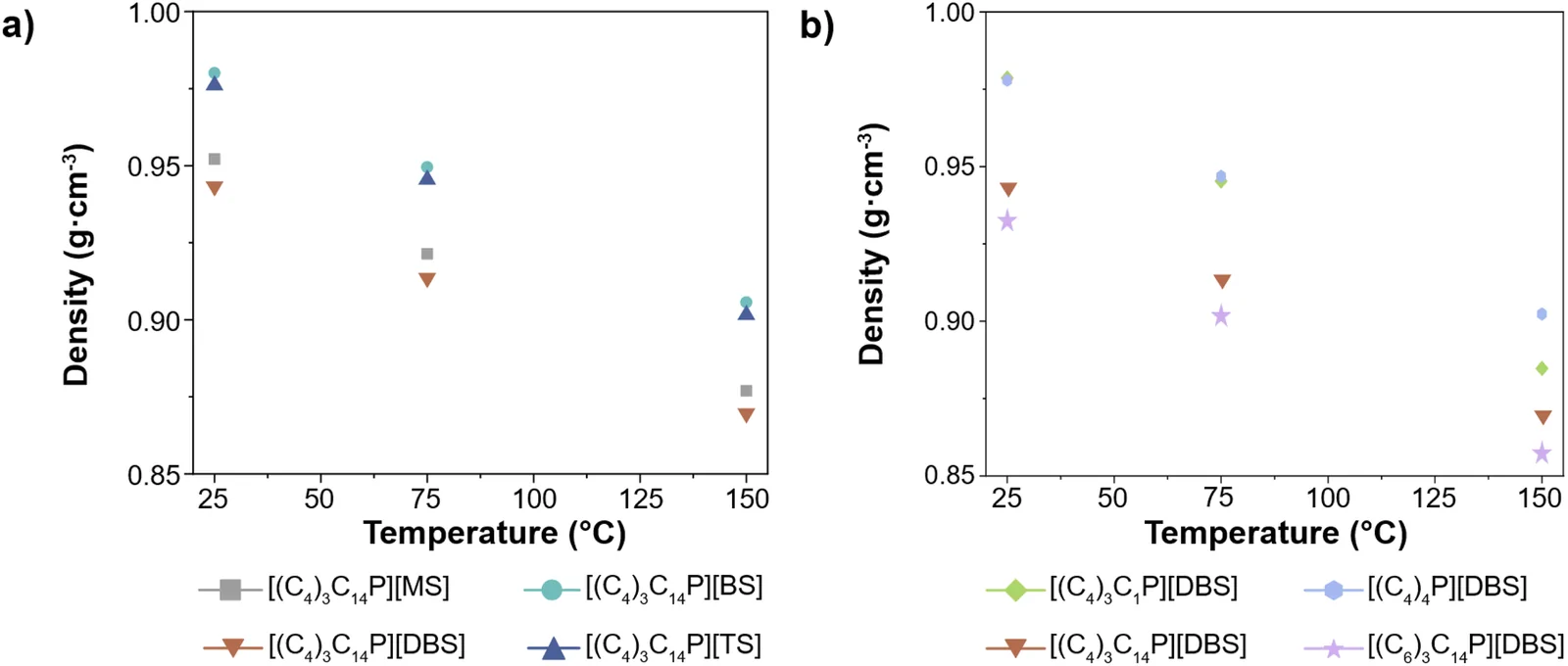
The dependence of density on temperature for (a) [(C_4_)_3_C_14_P]-based ILs and (b) [DBS]-based ILs at 25 °C, 75 °C and 150 °C.

The density of [(C_4_)_3_C_14_P]-based ILs (Fig. 4a) is primarily influenced by the nature of the anion. For ILs containing aromatic anions, density decreased with increasing alkyl chain length in the *para* position, following the order [(C_4_)_3_C_14_P][BS] > [(C_4_)_3_C_14_P][TS] > [(C_4_)_3_C_14_P][DBS]. A similar trend was observed by Panda *et al.* for pyrrolidinium-based ILs, who attributed this behavior to the increase in alkyl chain length, which reduces packing efficiency and, consequently, lowers density.^33^ In contrast, the lower density observed for the [MS] anion compared to [BS] systems may be attributed to differences in anion molecular weight, as reported for other IL families.^34–36^ When considering the effect of cation, a consistent trend was observed for [DBS]-based ILs at 25 °C (Fig. 3 and 4b) the density follows the order [(C_4_)_3_C_1_P][DBS] > [(C_4_)_4_P][DBS] > [(C_4_)_3_C_14_P][DBS] > [(C_6_)_3_C_14_P][DBS]. This indicates that density decreased with increasing cation alkyl chain length. This effect can be associated with the growth of the non-polar region upon increasing alkyl chain length, which results in an enhancement in molecular volume and a decrease in packing efficiency.^33,37^ Overall, these results show that increasing alkyl chain length leads to lower density because it reduces packing efficiency, indicating that the density of phosphonium-sulfonate ILs can be tuned through the rational design of cation–anion combinations. To further quantify the temperature dependence of the studied ILs density, the experimental data were fitted using eqn (1) where *ρ* is the density, *T* is the temperature in K and *a* and *b* are the fitting parameters (Table S2).^38^ Using eqn (1), excellent linear correlations were obtained for all studied ILs confirming that for all the ILs density decreases linearly with increasing temperature on the investigated temperature range. These linear correlations were used as an empirical description of the density-temperature behaviour within the investigated range (25–150 °C), where the effect of cation and anion structure remains consistent at the three measured temperatures. The fitted *a* values were similar for most of the ILs around 5.8–6.1 × 10^−4^ g cm^−3^ K^−1^ indicating comparable thermal expansion behavior within the studied IL family. The only slightly higher temperature coefficient was observed for [(C_4_)_3_C_1_P][DBS] although this IL also showed the largest experimental uncertainty at 150 °C (Table S2). These results indicate that even if the absolute density values are clearly influenced by the selected cation and anion structure, the temperature dependence of density remains predictable and approximately linear for all studied phosphonium-sulfonate ILs.

1 *ρ* = *b* − *aT*

#### 3.2.4. Viscosity

The viscosity (*η*) of ILs is strongly governed by ion structure and intermolecular interactions. As a key transport property, it plays an important role in mass transfer, fluid flow, and overall process performance, and is particularly important in the design of industrial processes involving heat and/or mass transfer, dissolution, and related operations.^36,39^ For phosphonium-sulfonate ILs, viscosity was measured at six different temperatures between 25 °C and 200 °C (Table S3). The measured values of viscosity as a function of temperature are depicted in Fig. 5 and detailed in Table S3. It is important to underline that the reported physicochemical properties correspond to the experimentally characterized samples and should not be interpreted as properties of strictly anhydrous ILs.

**Fig. 5:**
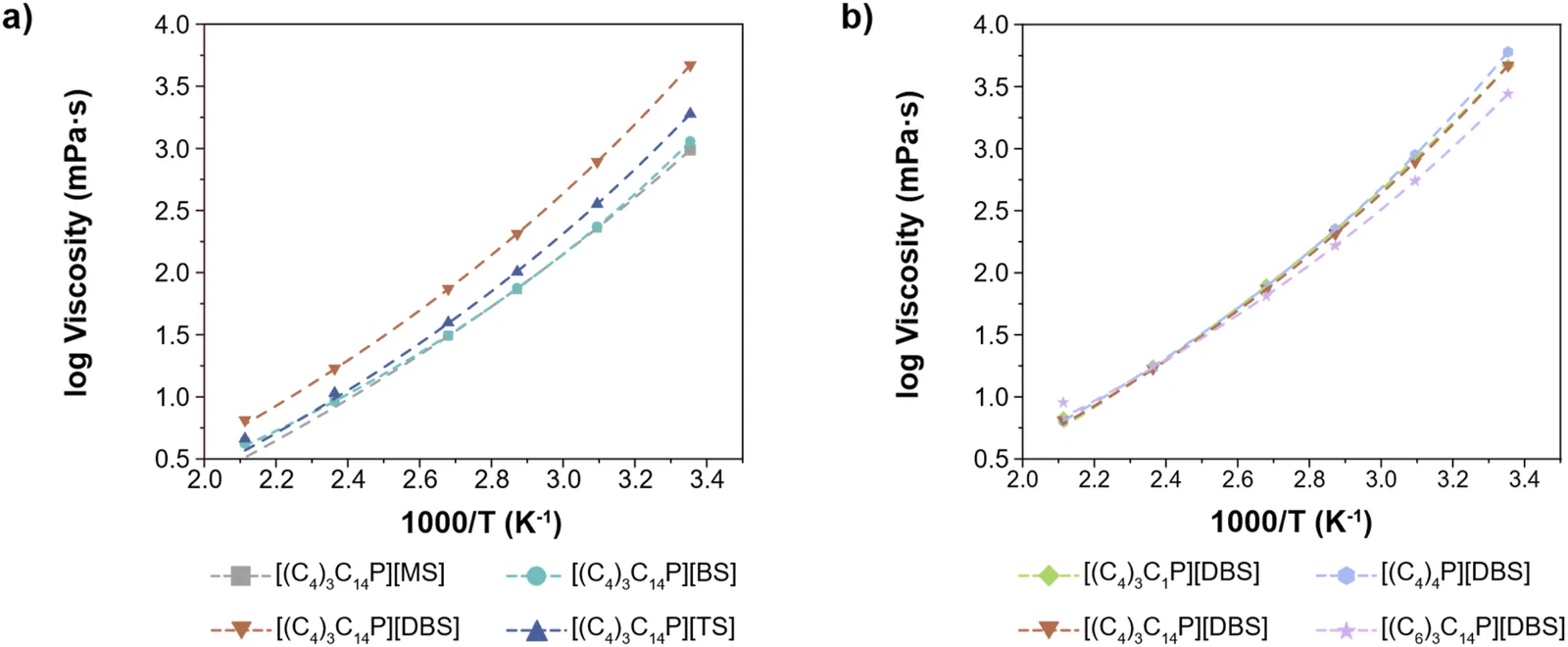
Temperature dependence of the logarithm of viscosity, represented as log_10_(*η*) *vs.* 1000/*T*, for (a) [(C_4_)_3_C_14_P]-based ILs and (b) [DBS]-based ILs. Symbols represent the experimental viscosity data measured at 25, 50, 75, 100, 150, and 200 °C, while dashed lines represent the corresponding VTF fits.

As commonly observed for ILs, viscosity decreased markedly with increasing temperature.^33,36,37,39,40^ This temperature dependence was particularly pronounced between 25 °C and 50 °C. For instance, the viscosity of [(C_4_)_3_C_14_P][DBS] decreased from approximately 4685 mPa s at 25 °C to about 781 mPa s at 50 °C. At higher temperatures, the decrease became less pronounced. This trend can be attributed to the weakening of cohesive intermolecular interactions with increasing temperature, which reduces resistance to flow.^37^ No simple structure–viscosity relationship was observed for the phosphonium-sulfonate ILs. At 25 °C, the viscosity followed the order: [(C_4_)_4_P][DBS] > [(C_4_)_3_C_14_P][DBS] > [(C_4_)_3_C_1_P][DBS] > [(C_6_)_3_C_14_P][DBS] > [(C_4_)_3_C_14_P][TS] > [(C_4_)_3_C_14_P][BS] > [(C_4_)_3_C_14_P][MS]. Although the overall structure–viscosity relationship is complex, ILs containing the [DBS] anion clearly exhibited the highest viscosity values.

The viscosity of [(C_4_)_3_C_14_P]-based ILs was primarily governed by the nature of the anion (Fig. 5a and Table S3). For ILs containing aromatic anions, viscosity increased with increasing alkyl chain length in the *para* position, following the order [(C_4_)_3_C_14_P][DBS] > [(C_4_)_3_C_14_P][TS] > [(C_4_)_3_C_14_P][BS]. This trend was opposite to that observed for density and, although it may appear counterintuitive, it was previously reported for other IL families. In these ILs, increasing the alkyl chain length enhances steric hindrance and van der Waals interactions, which reduce ion mobility and lead to higher viscosity.^37,41,42^ In contrast, the lower viscosity observed for the [MS]-based IL compared to the [BS] analogue may be attributed to the absence of an aromatic ring. The aromatic structure in the [BS] anion can promote additional π–π interactions, leading to stronger intermolecular interactions and a more rigid structure, which resulted in higher viscosity.^39^ In contrast to the anion effect, the cation influence within the [DBS]-based IL series was not systematic, and no clear structure–viscosity relationship could be established (Fig. 5b and Table S3). Similar observations were reported in the literature. For example, Tantray *et al.* noted the lack of a systematic trend in the viscosities of phosphonium- and choline-based ILs, suggesting that viscosity is not determined solely by ion size or molecular weight, but rather by a complex combination of cation–anion interactions.^37^ In this context, factors such as ion symmetry, charge distribution, and specific intermolecular interactions appear to play key roles in determining the overall viscosity. Similarly, Gaciño *et al.* also reported that the viscosity trend for each type of anion varies differently depending on the type of cation.^36^ These findings agree with previous studies on structure–viscosity relationships in ILs and highlight the complex interplay between cation and anion structures. In general, the size and structural complexity of the anion tend to increase viscosity, whereas the influence of the cation is less straightforward and depends on the specific cation–anion combination. In Fig. 5a and b the temperature dependent viscosity values show a clear curvature. This is important because it confirms that viscosity does not follow a simple Arrhenius behavior over the entire studied temperature range but rather a nonlinear Vogel–Fulcher–Tammann (VFT) type dependence. Consequently, the temperature dependence of viscosity was further analyzed using the VFT equation (eqn (2)), where *η*_0_, *B*_*η*_ and *T*_0,*η*_ are the fitting parameters.^43^ The VFT equation accurately described the experimental viscosity data within the studied 25–200 °C range, with *R*^2^ values close to unity for all ILs (Table 2). As observed in Table 2, the calculated fragility parameters, *δ*_*η*_ = *B*_*η*_/*T*_0_ ranged from 7.7 to 15.0 indicating fragile-liquid behavior and confirming the non-Arrhenius nature of viscous flow for this family of ILs.^44^ Apparent activation energies (*E*_a,*η*_) for viscous flow were estimated from Arrhenius plots of ln(*η*) *vs.* 1/*T* in the 25–50 °C range where the decrease in viscosity was more evident. The obtained *E*_a,*η*_ values (Table 2) ranged from 46 to 61 kJ mol^−1^, with the highest value of apparent activation energy observed for [(C_4_)_4_P][DBS] IL. In general, [DBS]-based ILs showed higher apparent activation energies than the [MS]-, [BS]-, and [TS]-based analogues, supporting the conclusion that the bulky [DBS] anion increases the energetic barrier for ion rearrangement during viscous flow.

2 *η* = *η*_0_exp[*B*_*η*_/(*T* − *T*_0,*η*_)]

**Table 2:** VFT equation parameters (*η*_0_, *B*_*η*_, *T*_0,*η*_), fragility parameter (*δ*_*η*_) and local apparent activation energies for viscous flow (*E*_a,*η*_) calculated from viscosity data. The *R*^2^ values indicate the goodness of fitting

IL	*η*_0_ (mPa s)	*B*_*η*_ (K)	*T*_0,*η*_ (K)	*R* ^2^	*δ*_*η*_	*E*_a,*η*_ (kJ mol^−1^)
[(C_4_)_3_C_14_P][MS]	0.014 ± 0.007	1869 ± 193	131 ± 10	0.99999	14.3	46
[(C_4_)_3_C_14_P][BS]	0.060 ± 0.004	1290 ± 22	167 ± 1	1.00000	7.7	51
[(C_4_)_3_C_14_P][TS]	0.020 ± 0.005	1675 ± 79	152 ± 4	1.00000	11.0	54
[(C_4_)_3_C_14_P][DBS]	0.025 ± 0.002	1757 ± 21	154 ± 1	1.00000	11.4	57
[(C_4_)_3_C_1_P][DBS]	0.012 ± 0.003	2061 ± 76	138 ± 3	1.00000	15.0	55
[(C_4_)_4_P][DBS]	0.037 ± 0.005	1587 ± 41	166 ± 2	1.00000	9.6	61
[(C_6_)_3_C_14_P][DBS]	0.048 ± 0.009	1586 ± 58	154 ± 3	1.00000	10.3	52

Therefore, although phosphonium-sulfonate ILs exhibit high viscosities at room temperature, the VFT analysis confirms that their viscosity decreases strongly and predictably with temperature, reaching values below 10 mPa s at 200 °C for all systems (Table S3). This pronounced temperature response is relevant for assessing their potential in thermally demanding applications, as increasing temperature substantially reduces the limitations associated with their high room-temperature viscosity.

## 4. Assessment of temperature ranges over which the ILs remain liquid and thermally stable

The potential of a given IL for use under thermally demanding conditions can be assessed from the temperature range over which it remains liquid and exhibits limited mass loss under defined thermal-stability testing conditions. In this context, the estimated temperature range over which the IL remains liquid and thermally stable can be defined by a lower limit associated with the melting temperature (*T*_m_) and an upper limit determined by isothermal performance. The gap between the melting point and decomposition temperatures observed for the studied phosphonium-sulfonate ILs places this IL family among the promising candidates for a wide range of thermal fluid applications. Fig. 6 shows the estimated liquid-phase operating range for each IL, defined here from the lower temperature limit associated with liquid-state behaviour to the upper temperature selected for the isothermal stability screening.

**Fig. 6:**
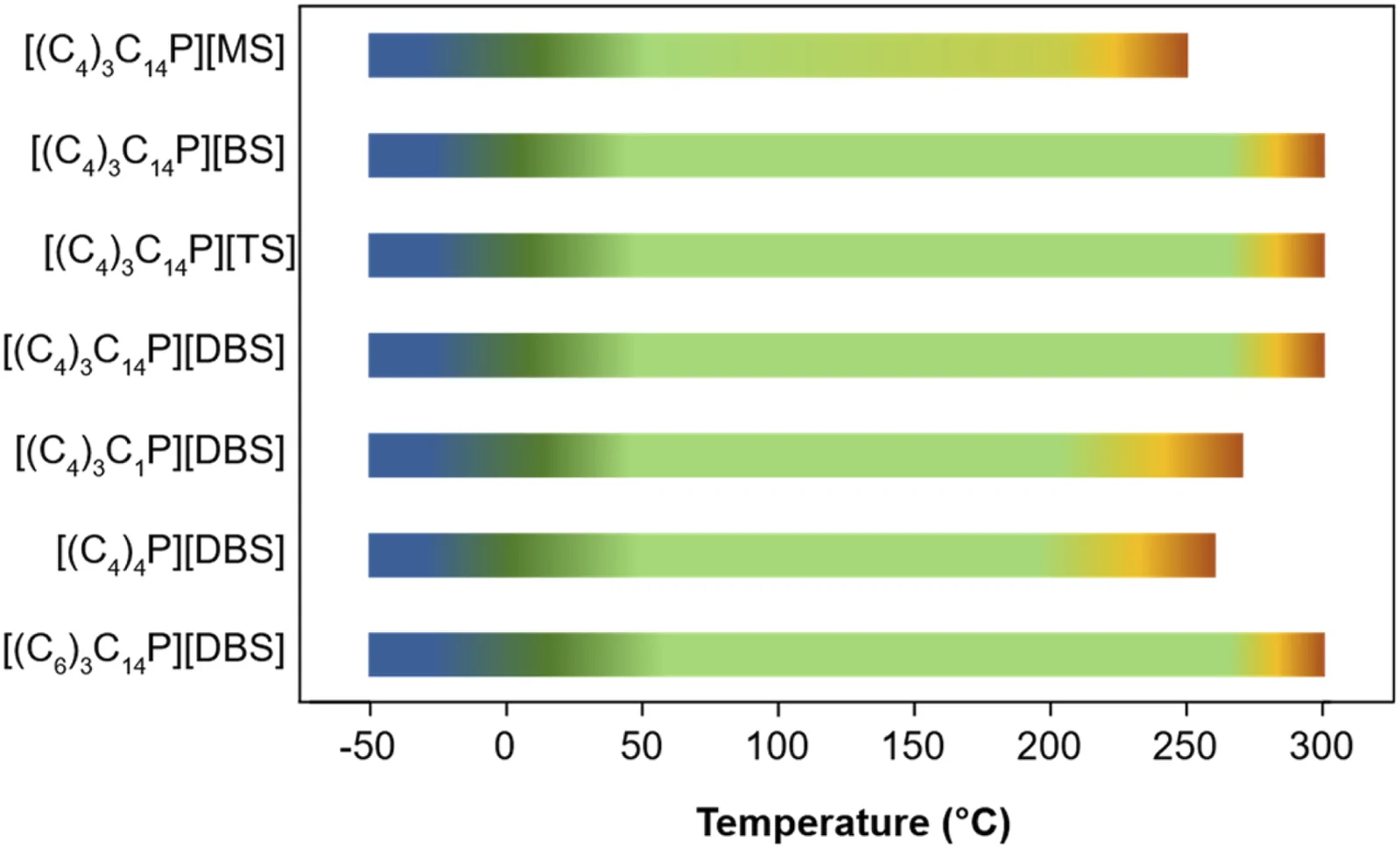
Estimated temperature ranges of the studied phosphonium-sulfonate ILs based on melting behaviour, thermal stability, and viscosity. The colour gradient qualitatively represents the transition from the low-temperature, high-viscosity region (blue), through the favourable liquid operating region (green), to temperatures progressively approaching the upper thermal stability limit considered for each IL (yellow to orange-red).

The lower temperature limits (blue and dark green) shown in Fig. 6 were established from the combined analysis of DSC (Fig. S6) and viscosity. More specifically, the melting peak temperature (*T*_peak_) was used as the main criterion, together with the viscosity behaviour at low temperature. As all the ILs remained liquid at room temperature (Table 1 and Fig. S6), the studied systems can be considered to exhibit a broad liquid-phase temperature range, which is desirable for applications requiring thermally stable fluids over a wide temperature range. Additionally, as discussed above, the practical applicability of these fluids must also consider their degradation behaviour. If only *T*_5%_ values are considered, all the ILs exhibit *T*_5%_ close to 400 °C. However, as previously discussed, isothermal studies provide a more realistic evaluation of thermal stability under practical conditions. In this work, 300 °C was considered as the upper temperature limit. At this temperature, four of the ILs ([(C_4_)_3_C_14_P][TS], [(C_4_)_3_C_14_P][DBS], [(C_4_)_3_C_14_P][BS] and [(C_6_)_3_C_14_P][DBS]) showed less than 5% weight loss after 12 h, indicating good thermal stability. Therefore, for these ILs, the preliminary liquid-phase operating range considered in this work can be defined from the temperature at which they exhibit liquid-state behaviour up to 300 °C.

In addition to melting temperature and thermal stability, viscosity is another important property to consider when assessing the practical applicability of these ILs as high-temperature fluid candidates. The studied phosphonium-sulfonate ILs showed relatively high viscosities at low temperature, ranging from 971.5 to 6021.2 mPa s at 25 °C. However, viscosity decreased strongly with increasing temperature, reaching 73.2–224.6 mPa s at 75 °C and 4.2–9.0 mPa s at 200 °C. This pronounced reduction indicates that moderate heating substantially improves the handling characteristics of these ILs. Therefore, the temperature range above 50–75 °C can be considered a more favourable handling range for applications where reduced viscosity is required, while operation at higher temperatures further mitigates the limitations associated with high room-temperature viscosity. Overall, the phosphonium-sulfonate ILs exhibited a broad temperature range over which they remained liquid and thermally stable, together with decreasing viscosity at higher temperatures, supporting their potential for thermally demanding applications. The temperature range most suitable from a fluidity perspective was that in which the ILs remained liquid and exhibited sufficiently low viscosity for practical handling, generally above 50–75 °C. This range may therefore be considered a favorable temperature interval from a viscosity perspective, based on the criterion adopted in this study. However, when suitable equipment is available to operate with more viscous fluids, the practical use of these ILs can be extended beyond this favorable range.

In addition to the temperature range over which an IL remains liquid and thermally stable, the duration for which it can maintain this stability at a given temperature is also an important parameter for assessing its practical potential. Therefore, to provide a more robust assessment of isothermal stability, [(C_4_)_3_C_14_P][DBS] was selected as a representative IL and subjected to isothermal experiments at 250, 275, 300, 325, and 350 °C (SI S5). The resulting isothermal data were then used to develop a stability estimation approach based on the *T*_0.01/10 h_ criterion, defined as the temperature at which a 1 wt% mass loss is predicted after 10 h under the selected atmosphere and experimental conditions.^45^ The approach can also predict the time required to reach different mass-loss levels at a given temperature.

As shown in Fig. 7, a series of isothermal thermogravimetric analyses were performed at different temperatures for 12 h for the [(C_4_)_3_C_14_P][DBS]. Mass losses were relatively low over most of the temperature range investigated, except at 350 °C, where the isothermal mass loss reached approximately 45%. At some temperatures, an initial non-linear region was observed during the first 2–3 h, which may be associated with the loss or degradation of minor residual species. After this, mass loss followed an approximately linear trend with time. To ensure that only the clear linear region was considered at all temperatures, the linear fits were performed using the data recorded from 6 h onwards. These equations were then used to estimate the mass loss at a given time, or the time required to reach a given mass loss level.

**Fig. 7:**
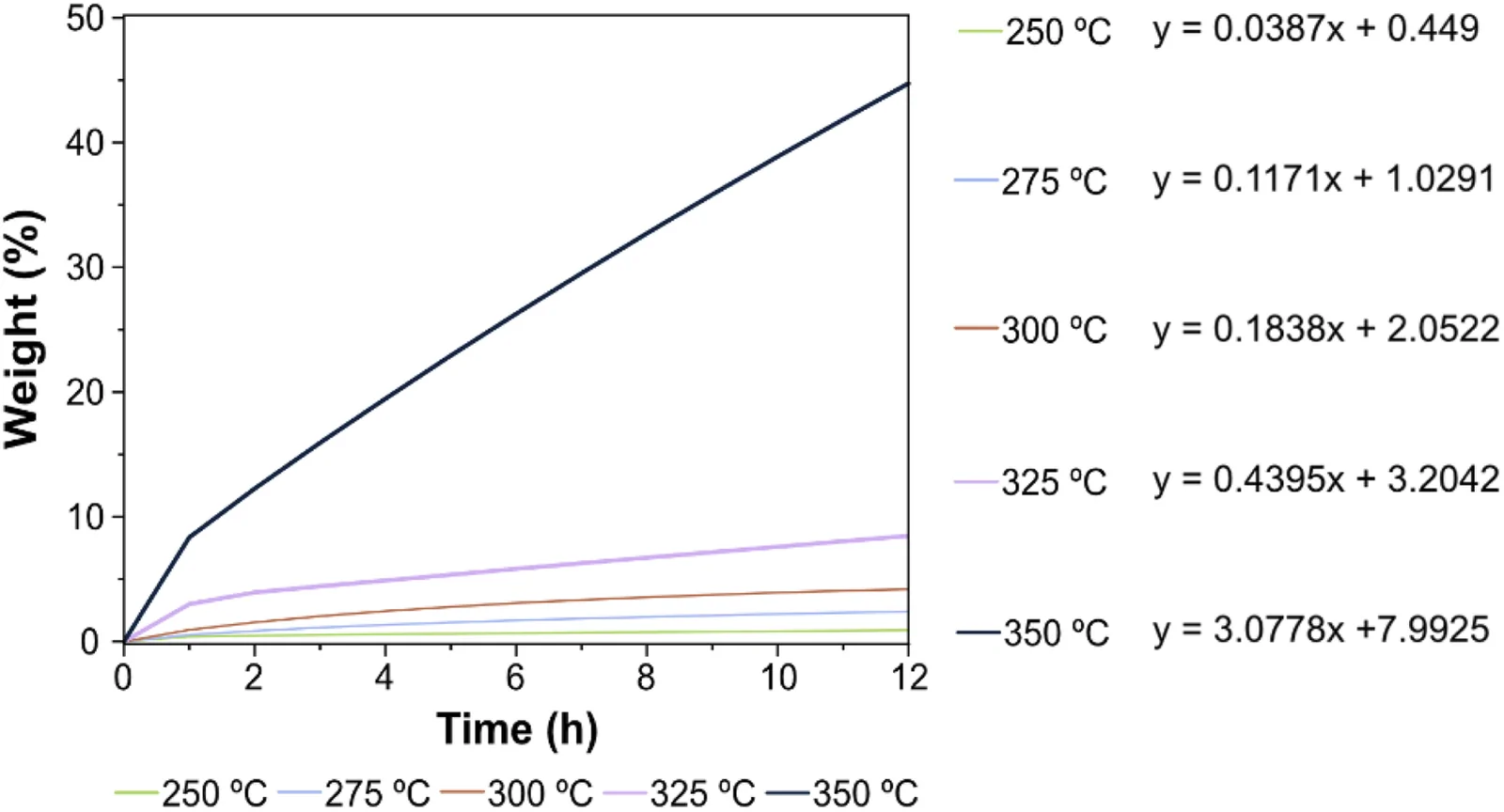
Isothermal thermal stability of [(C_4_)_3_C_14_P][DBS]at different temperatures.

Using these linear equations, the time required to reach 1% and 2% mass loss was calculated at each temperature (Fig. 8). The resulting values followed an exponential trend with temperature, allowing empirical equations to be derived to estimate the time required to reach 1% or 2% mass loss at any given temperature within the studied range. Conversely, this relationship can also be used to estimate the maximum temperature theoretically compatible with a predefined exposure time and mass-loss threshold. For example, for an exposure time of 10 h, the estimated temperatures corresponding to 1% and 2% mass loss were 279 and 296 °C, respectively. Similarly, at 250 °C, the estimated times to reach 1% and 2% mass loss were 31.4 and 62.9 h, respectively. These calculations provide a useful predictive tool to compare the relative thermal robustness of the ILs under prolonged heating.

**Fig. 8:**
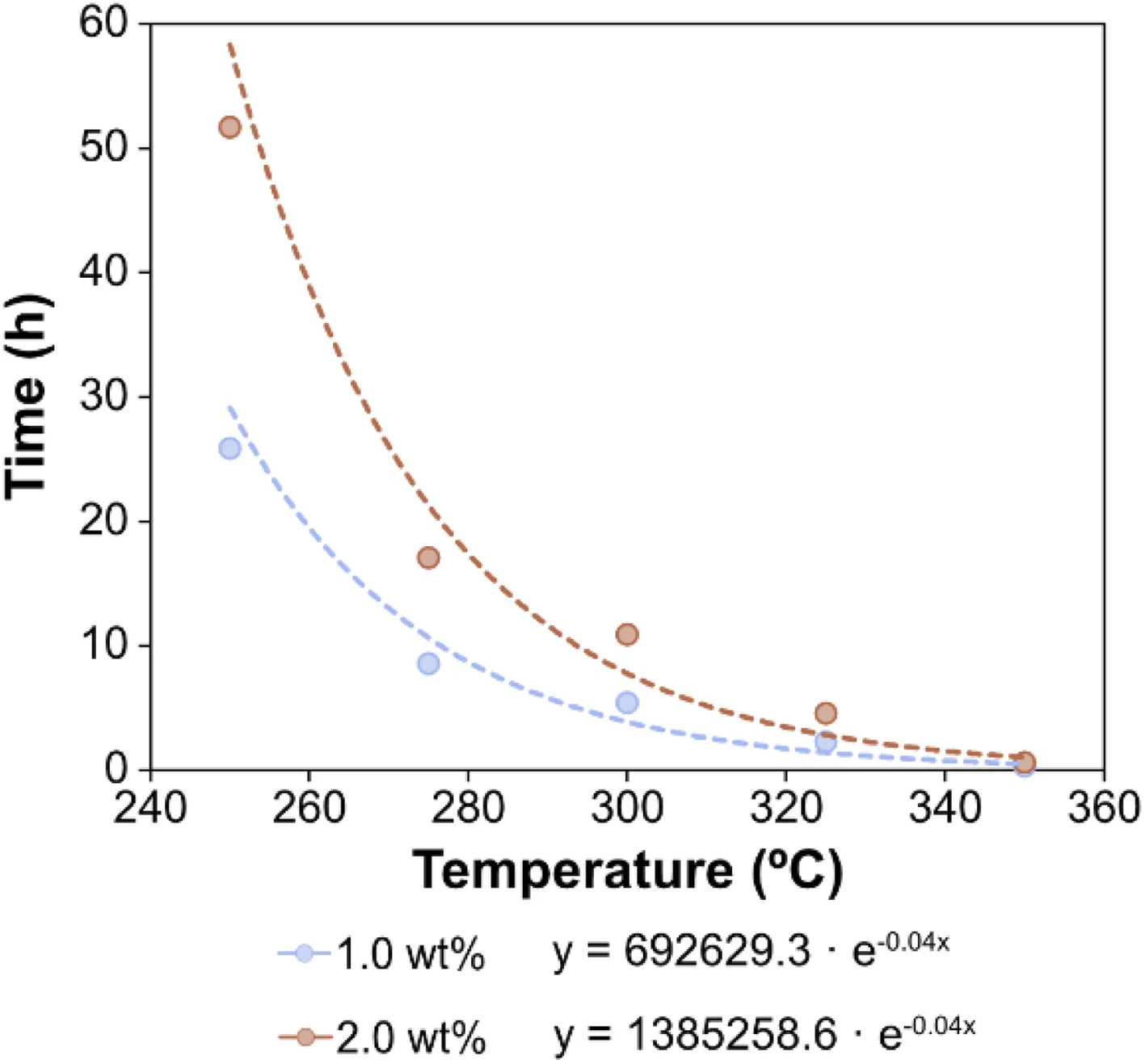
Weight loss rates for 1 wt% and 2 wt% at different temperatures for [(C_4_)_3_C_14_P][DBS].

## 5. Conclusions

A series of non-fluorinated phosphonium-sulfonate ILs was synthesized, characterized, and systematically investigated to establish structure–property relationships relevant to their potential use under elevated-temperature conditions. All ILs exhibited high thermal stability under dynamic conditions, with *T*_5%_ values between 385 and 406 °C. However, isothermal measurements showed that dynamic thermal parameters may overestimate practical stability, confirming the need to evaluate thermal robustness under prolonged exposure at the target temperature. At 300 °C, four of the seven ILs showed weight losses equal to 2.6 wt% after 12 h, identifying them as the most promising systems within the prepared series of ILs. Thermal stability was strongly affected by ion structure. Within the [(C_4_)_3_C_14_P] cation-based series, aromatic sulfonate anions improved stability compared with the non-aromatic methanesulfonate analogue, whereas [DBS] anion-based ILs showed a less systematic but relevant cation-dependent behavior under isothermal conditions. Density and viscosity were also influenced by ionic liquid structure and temperature. Density decreased linearly with increasing temperature and alkyl-chain length, reflecting thermal expansion and reduced packing efficiency. In contrast, viscosity decreased strongly with temperature and increased with alkyl chain length within the aromatic-anion subseries, whereas the cation effect was not systematic. The VFT analysis confirmed non-Arrhenius viscosity behavior over the studied temperature range (25–200 °C) with all systems reaching viscosities below 10 mPa s at 200 °C.

By integrating melting behavior, isothermal thermal stability, density, viscosity, and viscosity-temperature modelling, this study defines the temperature ranges over which non-fluorinated phosphonium-sulfonate ILs remain liquid and thermally stable, extending up to 300 °C under the selected stability criterion. For [(C_4_)_3_C_14_P][DBS], additional isothermal measurements established empirical relationships between temperature, exposure time, and mass loss, enabling the prediction of degradation limits under different thermal exposure conditions. The results demonstrate that these ILs constitute a tunable platform in which thermal stability and transport properties can be adjusted through structure-guided cation–anion selection. This combined thermal and rheological assessment supports their consideration as candidate fluids for thermally demanding applications and provides selection criteria for further evaluation under application-specific conditions.

## Conflicts of interest

There are no conflicts to declare.

## Supplementary Material

RA-OLF-D6RA08417F-s001

## Data Availability

The data supporting this article have been included as part of the supplementary information (SI). Data for this article will be available at Zenodo repository at https://doi.org/10.5281/zenodo.23535680. Supplementary information is available. See DOI: https://doi.org/10.1039/d6ra08417f.
