# Supplementary material for: Non-fluorinated phosphonium-sulfonate ionic liquids as candidates for thermally demanding applications: thermal stability, viscosity and liquid-state temperature ranges
Source: RSC Adv. 2026 Nov 4. Online ahead of print. doi: 10.1039/d6ra08417f (PMC13732726; doi:10.1039/d6ra08417f)
Supplement: Supplementary file 1 [file RA-OLF-D6RA08417F-s001.pdf]

Supplementary Information (SI) for RSC Advances.
This journal is © The Royal Society of Chemistry

Non-fluorinated phosphonium-sulfonate ionic liquids as candidates for thermally demanding applications: thermal stability, viscosity and liquid-state temperature ranges

A. Centeno-Pedrazo,[1] J. Perez-Arce,[1,*] B. Iliev,[2] T.J.S. Schubert,[2] S. Lorenz,[2] A. Agrelo-Lestón,[1] M. Haumann,[3] M. Distaso,[3,4] C. Len,[5] I. Zabala,[6] S. Doppiu,[1] E.J. Garcia-Suarez,[1,7,*]

[1]Center for Cooperative Research on Alternative Energies (CIC energiGUNE), Basque Research and Technology Alliance (BRTA), Alava Technology Park, Albert Einstein 48, 01510 Vitoria-Gasteiz, Spain.

[2]IOLITEC Ionic Liquids Technologies GmbH, Salzstrasse 184, 74076, Heilbronn, Germany

[3]Friedrich-Alexander-Universität Erlangen-Nürnberg (FAU), Lehrstuhl für Chemische Reaktionstechnik (CRT), Egerlandstr. 3, 91058 Erlangen, Germany.

[4]Helmholtz-Institut Erlangen-Nürnberg for Renewable Energy (IET-2), Forschungszentrum Jülich GmbH, Cauerstraße 1, Erlangen91058, Germany.

[5]Chimie ParisTech, PSL Research University, CNRS, Institute of chemistry for Life and Health Sciences, 11 rue Pierre et Marie Curie, F-75005 Paris, France.

[6]SENER Ingeniería y Sistemas, S.A. Zugatzarte Etorb. 56, 48930 Getxo, Spain.

[7]IKERBASQUE, Basque Foundation for Science, Plaza Euskadi 5, 48009 Bilbao, Spain.

*corresponding authors: esuarez@cicenergigune.com; jperez@cicenergigune.com

Table of contents

Figure S1. General synthesis route for the studied phosphonium-sulfonate ionic liquids.

Figure S2. ^{1}H, $^{13}C\{^{1}H\}$ and $^{31}P\{^{1}H\}$ NMR spectra of the studied phosphonium-sulfonated ionic liquids: **a**) $[(C_4)_3C_{14}P][MS]$, **b**) $[(C_4)_3C_{14}P][BS]$, **c**) $[(C_4)_3C_{14}P][TS]$, **d**) $[(C_4)_3C_{14}P][DBS]$, **e**) $[(C_4)_3C_1P][DBS]$, **f**) $[(C_4)_4P][DBS]$, **g**) $[(C_6)_3C_{14}P][DBS]$.

Figure S3. TG profiles of the studied phosphonium-sulfonated ionic liquids: **a**) $[(C_4)_3C_{14}P][MS]$, **b**) $[(C_4)_3C_{14}P][BS]$, **c**) $[(C_4)_3C_{14}P][TS]$, **d**) $[(C_4)_3C_{14}P][DBS]$, **e**) $[(C_4)_3C_1P][DBS]$, **f**) $[(C_4)_4P][DBS]$, **g**) $[(C_6)_3C_{14}P][DBS]$.

Figure S4. Isothermal TG profiles of $[(C_4)_3C_{14}P][DBS]$ at 250 °C, 275 °C, 300 °C, 325 °C, and 350 °C.

Figure S5. ^{1}H and $^{31}P\{^{1}H\}$ NMR spectra of a) $[(C_4)_4P][DBS]$ and b) $[(C_4)_3C_{14}P][DBS]$ before and after isothermal treatment at 300 °C for 12 h.

Figure S6: DSC profiles of the studied phosphonium-sulfonated ionic liquids: **a**) $[(C_4)_3C_{14}P][MS]$, **b**) $[(C_4)_3C_{14}P][BS]$, **c**) $[(C_4)_3C_{14}P][TS]$, **d**) $[(C_4)_3C_{14}P][DBS]$, **e**) $[(C_4)_3C_1P][DBS]$, **f**) $[(C_4)_4P][DBS]$, **g**) $[(C_6)_3C_{14}P][DBS]$.

Table S1. Water and chloride content of the studied phosphonium-sulphonate ionic liquids determined by Karl Fischer titration.

Table S2. Density and linear fitting parameters at 25 °C, 75 °C and 150 °C of the studied phosphonium-sulfonated ionic liquids.

Table S3. Viscosity at 25 °C, 50 °C, 75 °C, 100 °C, 150 °C and 200 °C of the studied phosphonium-sulfonated ionic liquids.

Table S4. C, H, S, P and O theoretical and experimental values of the studied phosphonium-sulphonate ionic liquids determined by CHNS elemental analysis.

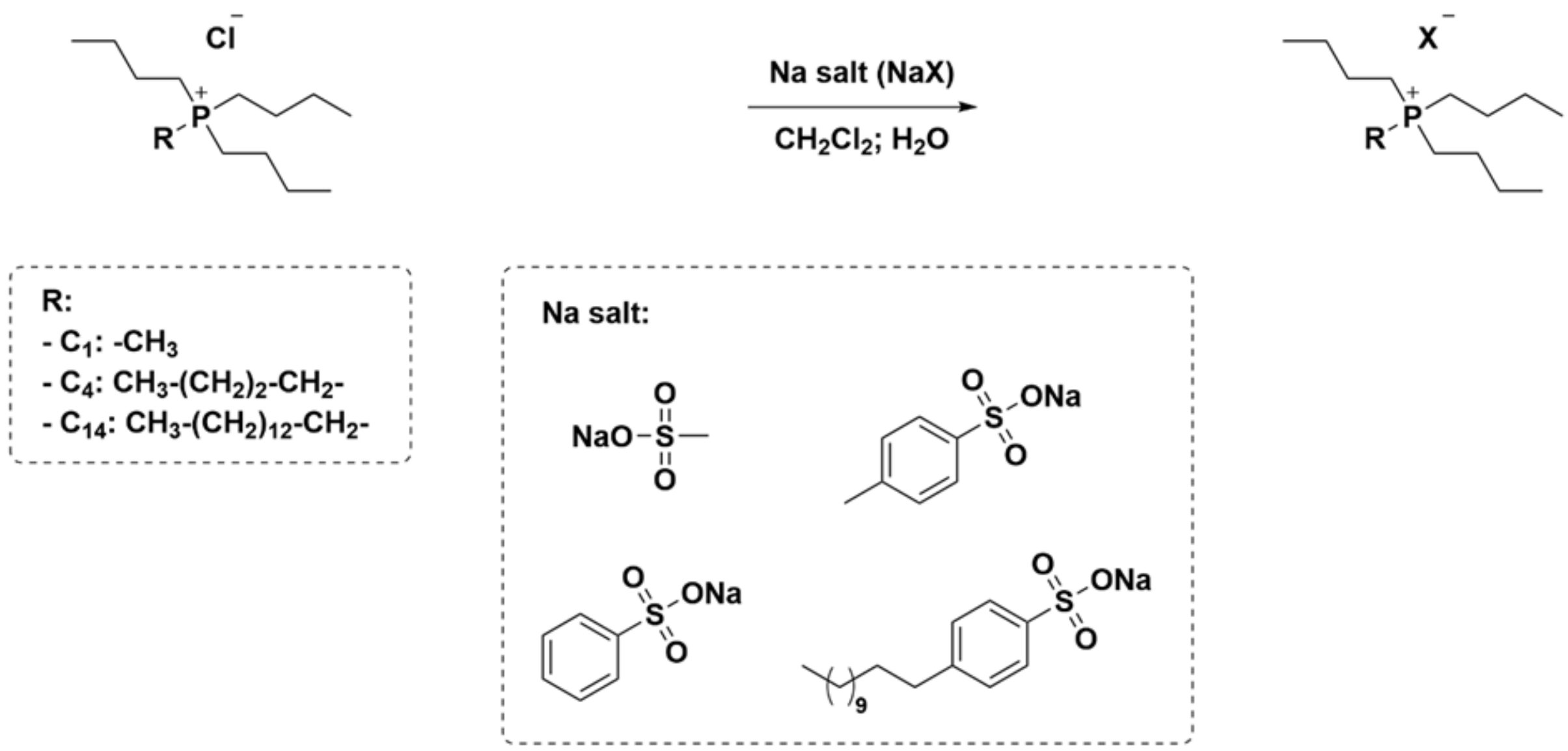

Figure S1. General synthesis route for the studied phosphonium-sulfonate ionic liquids.

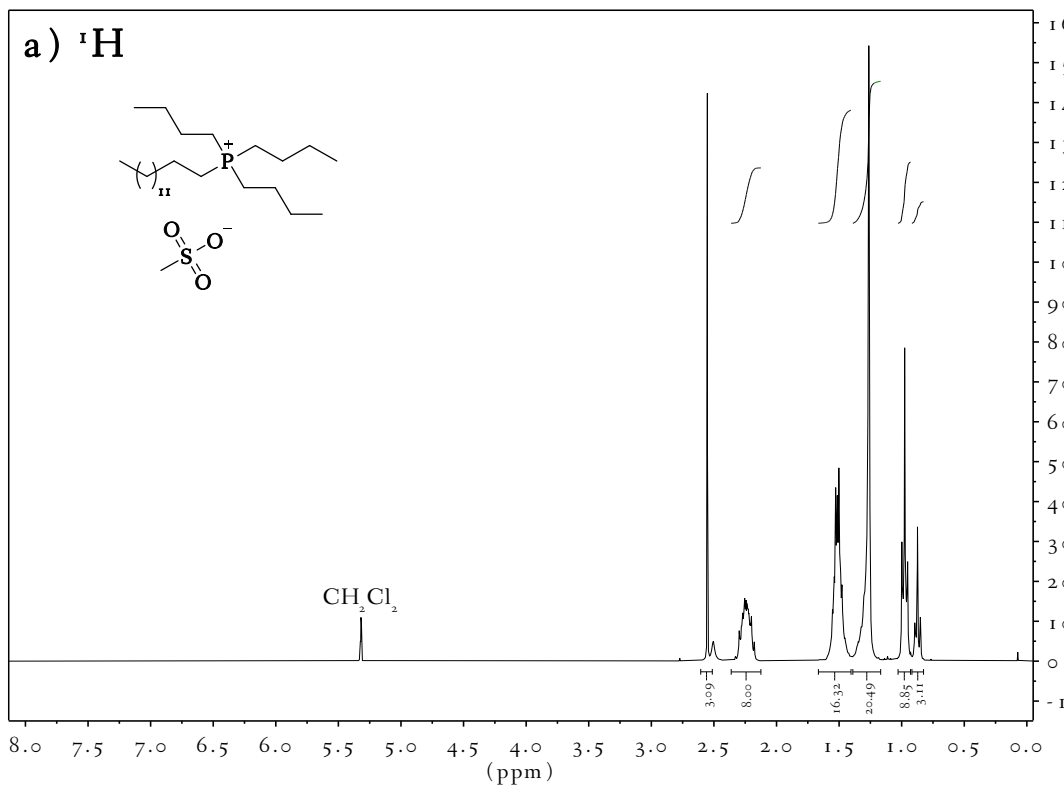

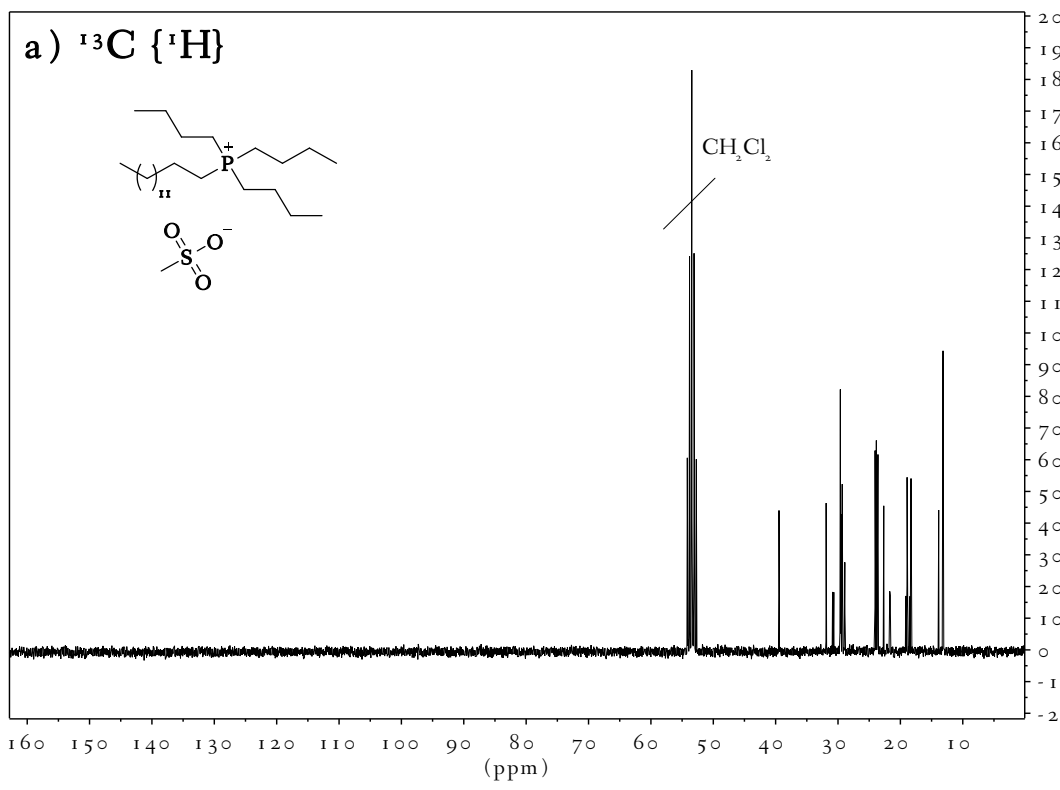

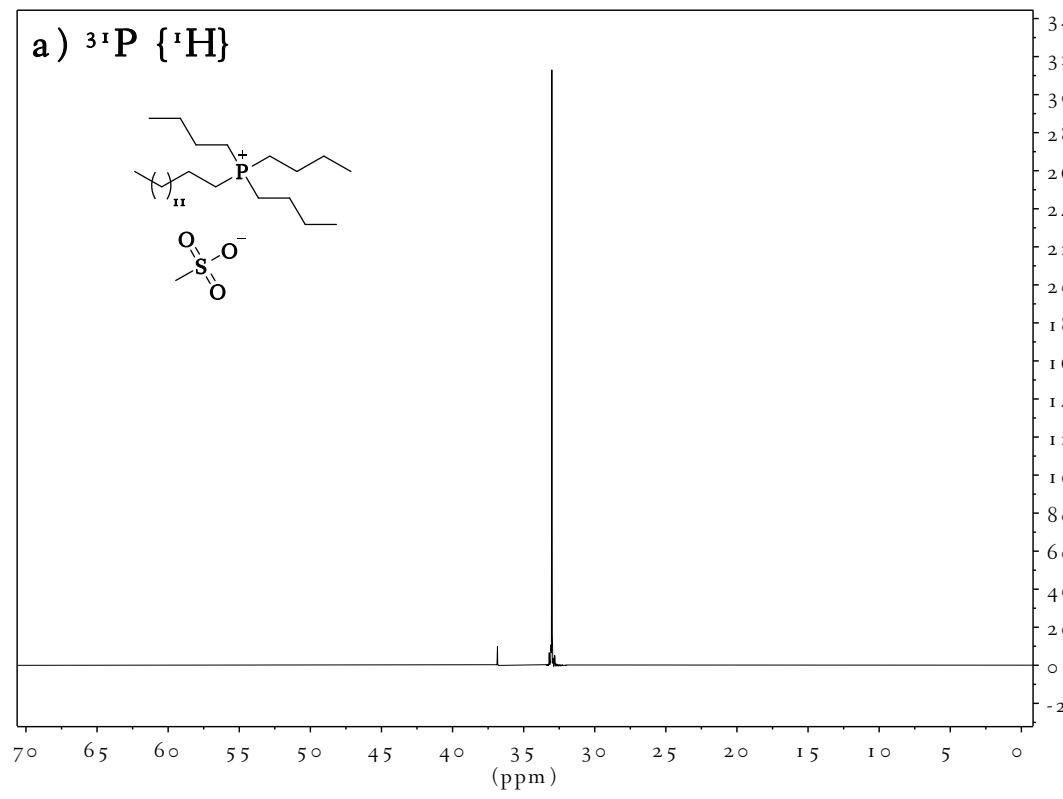

a) ^{1}H NMR (CH_2Cl_2-d_2, TMS, 300MHz) δ$_H$/ppm: 2.55 (s, 3H), 2.34-2.13 (m, 8H), 1.60-1.40 (m, 16H), 1.38-1.17 (m, 20H), 0.98 (t, 9H, J = 6.9 Hz), 0.87 (t, 3H, J = 6.6 Hz). ^{13}C {^{1}H} NMR (CH_2Cl_2-d_2, 75 MHz) δ$_C$/ppm: 39.46, 31.91, 30.87, 30.68, 29.66, 29.63, 29.60, 29.52, 29.33, 29.31, 28.90, 24.06, 23.86, 23.66, 23.60, 22.67, 21.70, 21.64, 19.15, 18.93, 18.53, 18.30, 13.86, 13.18. ^{31}P{^{1}H} NMR (CH_2Cl_2-d_2, 121 MHz) δ$_P$/ppm: 33.02

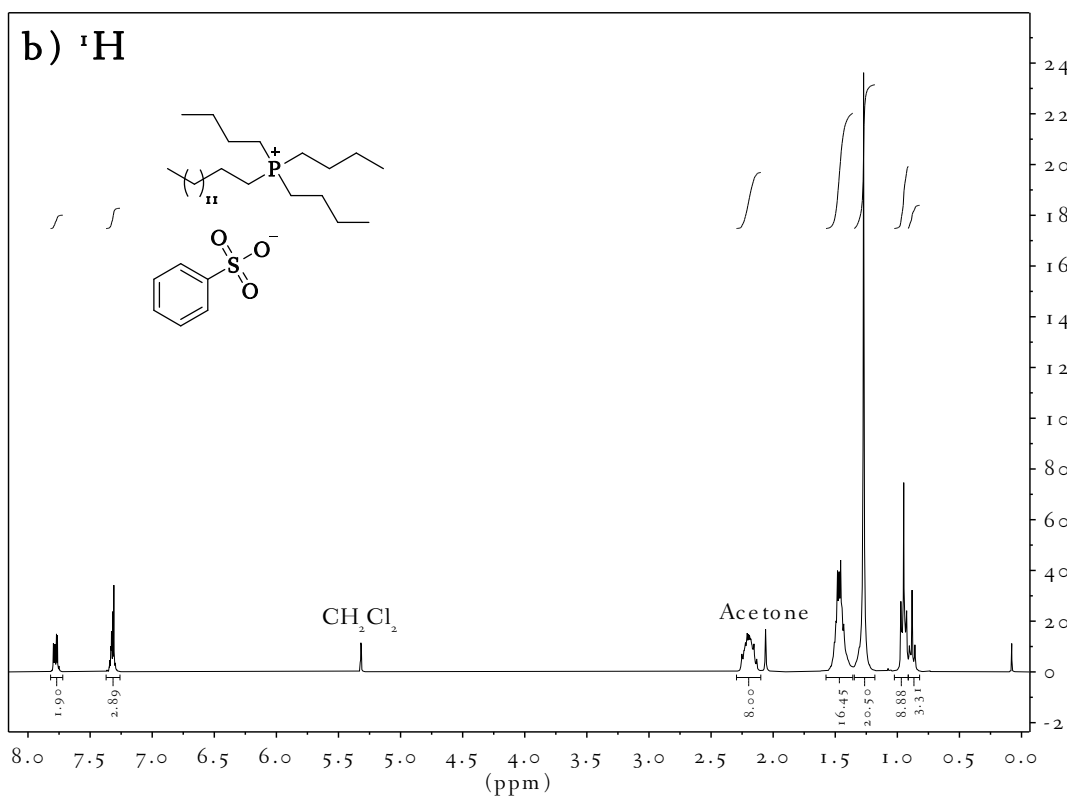

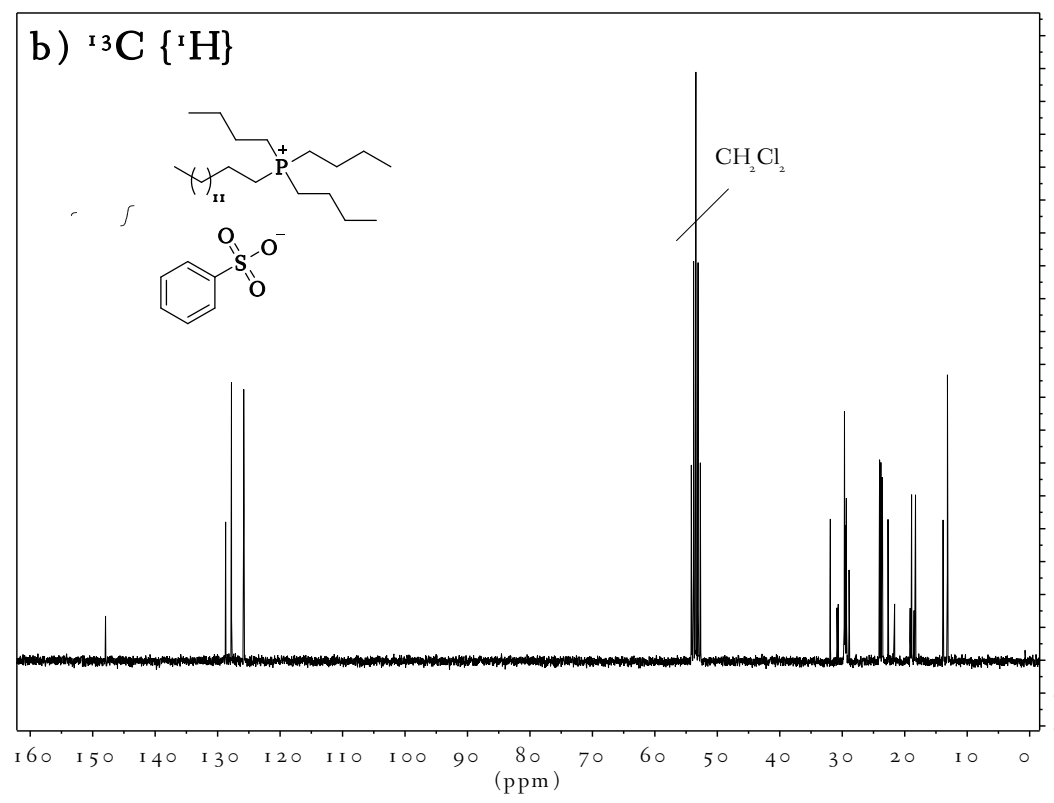

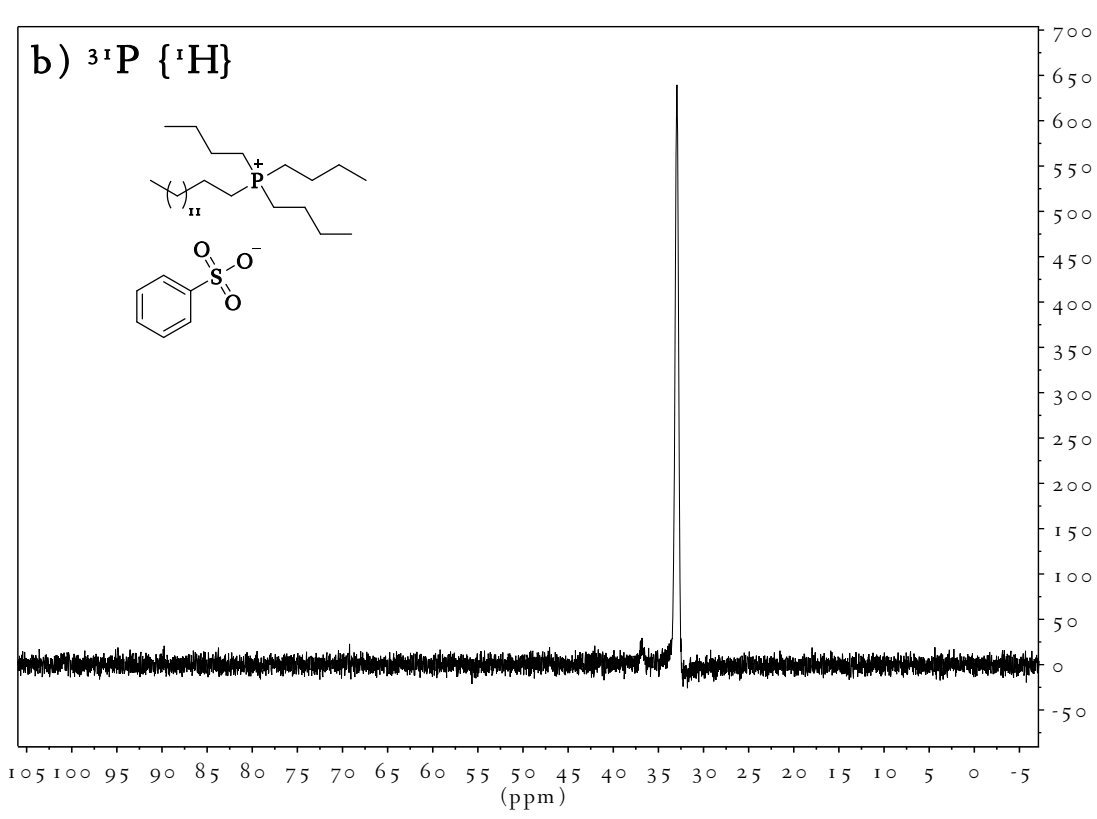

b) ^{1}H NMR (CH_2Cl_2-d_2, TMS, 300MHz) δ$_H$/ppm: 7.81-7.74 (m, 2H), 7.35-7.28 (m, 3H), 2.27-2.12 (m, 8H), 1.55-1.35 (m, 16H), 1.36-1.20 (m, 20H), 0.95 (t, 9H, J = 6.9 Hz), 0.88 (t, 3H, J = 7.5 Hz). ^{13}C {^{1}H} NMR (CH_2Cl_2-d_2, 75 MHz) δ$_C$/ppm: 147.94, 128.72, 127.78, 125.82, 31.92, 30.85, 30.65, 29.68, 29.65, 29.63, 29.53, 29.35, 29.33, 28.91, 24.03, 23.82, 23.64, 23.58, 22.68, 21.69, 21.63, 19.13, 18.91, 18.50, 18.28, 13.87, 13.17, 13.16. ^{31}P{^{1}H} NMR (CH_2Cl_2-d_2, 121 MHz) δ$_P$/ppm: 32.95.

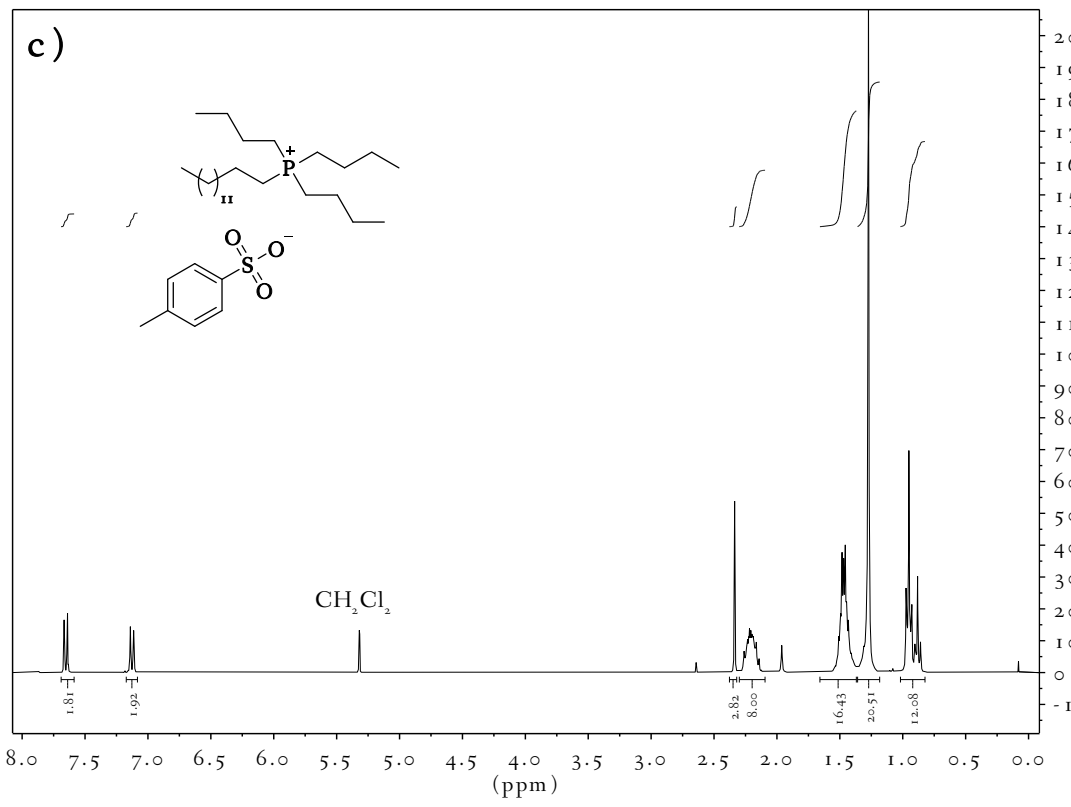

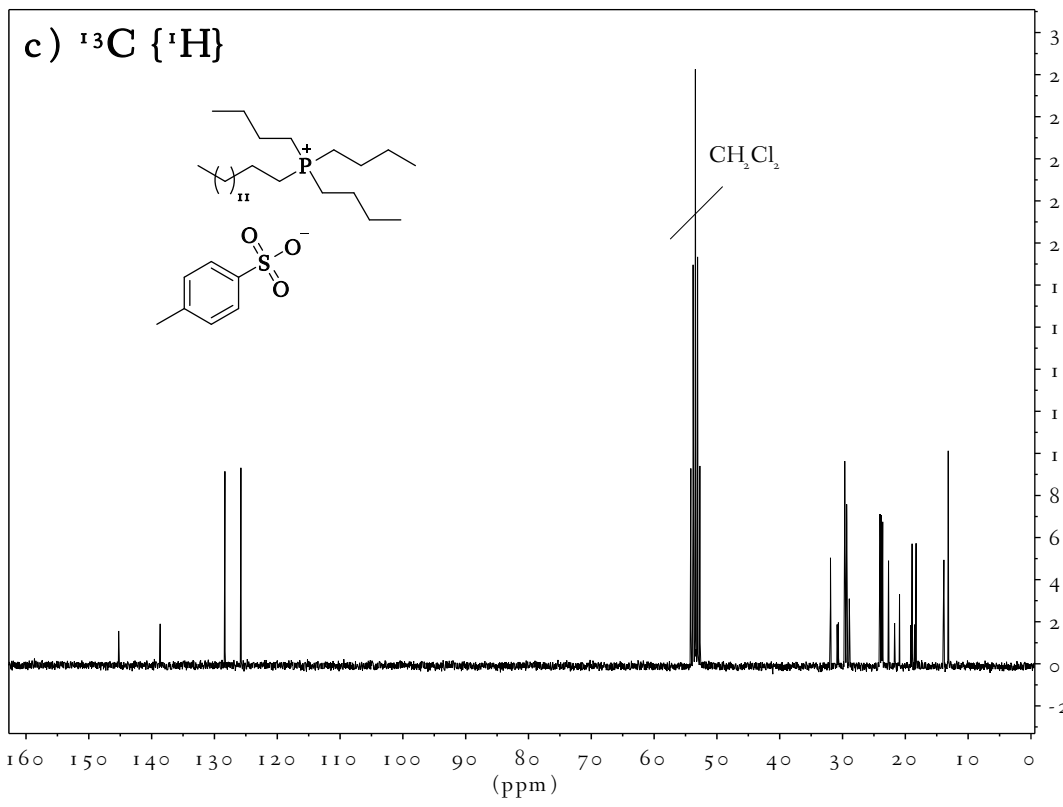

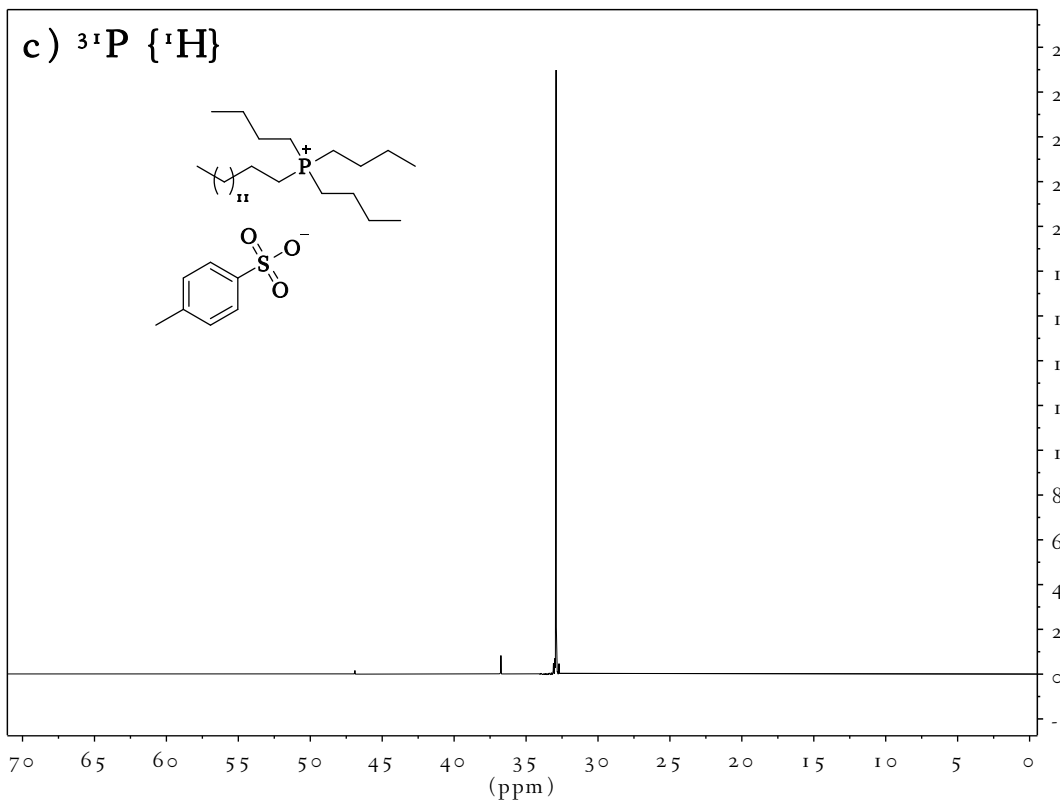

c) ^{1}H NMR (CH_2Cl_2-d_2, TMS, 300MHz) δ$_{H}$/ppm: 7.69-7.62 (m, 2H), 7.15-7.10 (m, 2H), 2.34 (s, 3H), 2.30-2.12 (m, 8H), 1.55-1.38 (m, 16H), 1.34-1.21 (m, 20H), 0.95 (t, 9H, J = 6.9 Hz), 0.88 (t, 3H, J = 6.9 Hz). ^{13}C {^{1}H} NMR (CH_2Cl_2-d_2, 75 MHz) δ$_{C}$/ppm: 145.25, 138.64, 128.33, 125.79, 31.92, 30.86, 30.66, 29.68, 29.66, 29.64, 29.54, 29.34, 28.92, 24.04, 23.83, 23.66, 23.60, 22.68, 21.71, 21.65, 20.93, 19.14, 18.92, 18.52, 18.29, 13.87, 13.17. ^{31}P{^{1}H} NMR (CH_2Cl_2-d_2, 121 MHz) δ$_{P}$/ppm: 32.92.

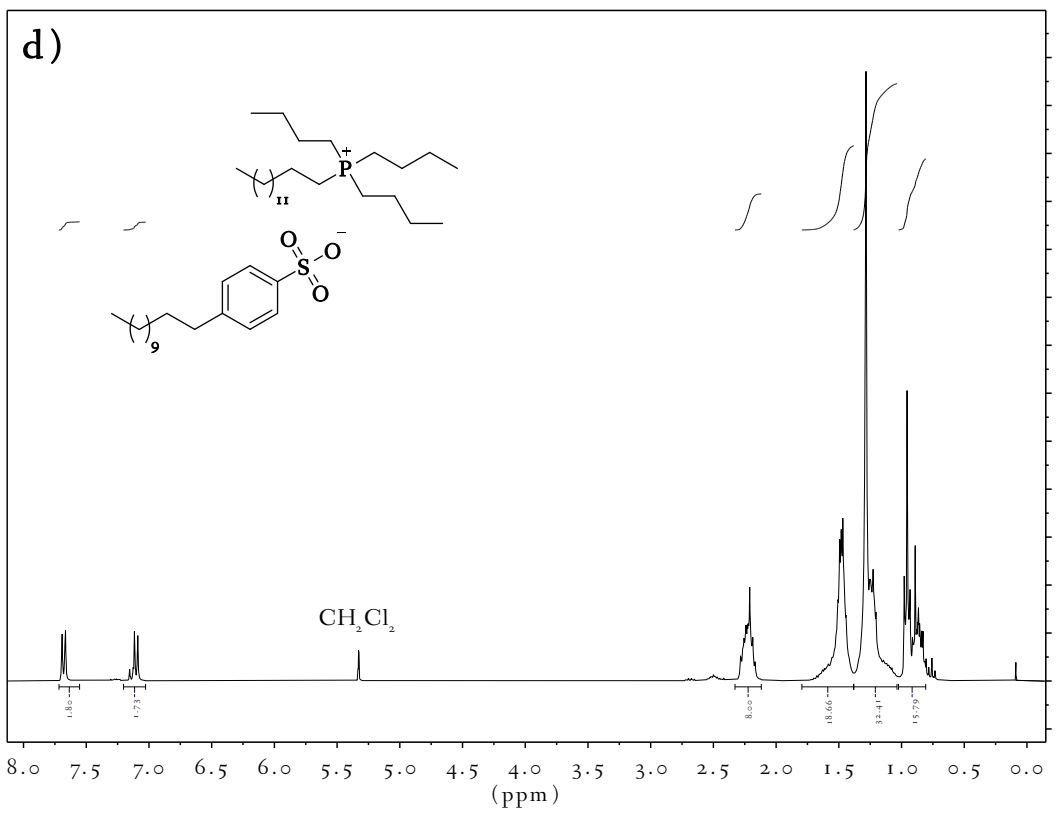

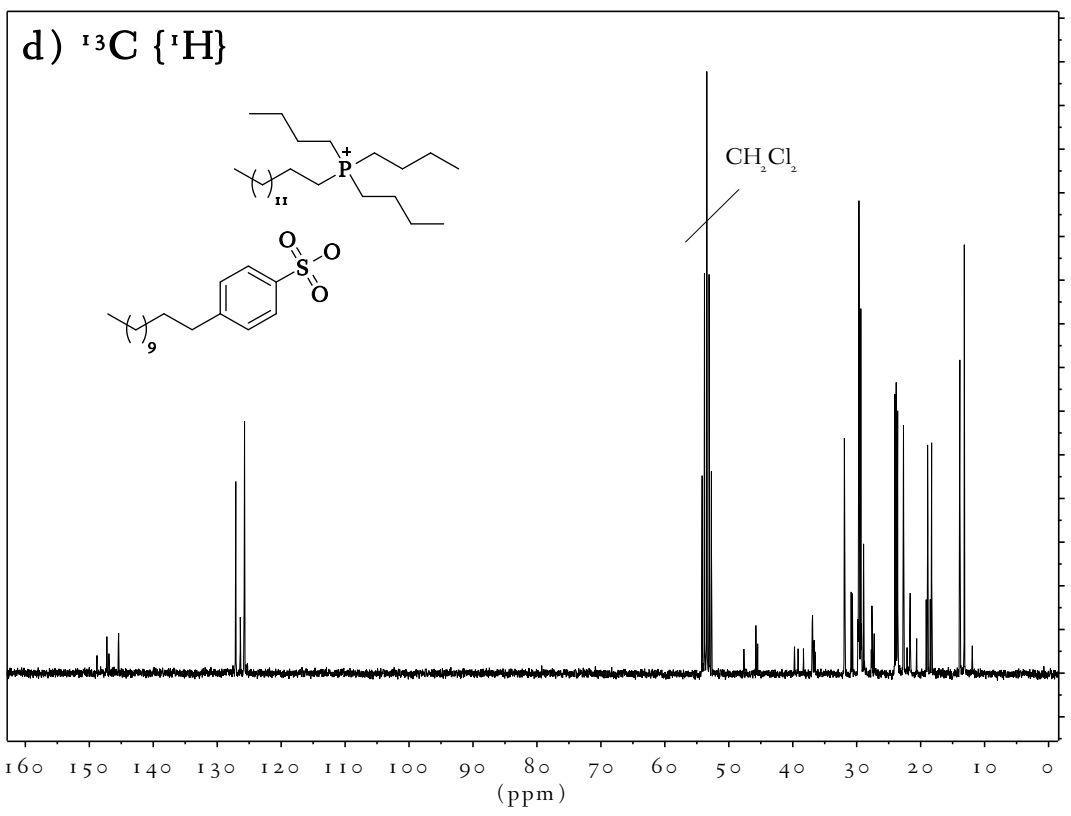

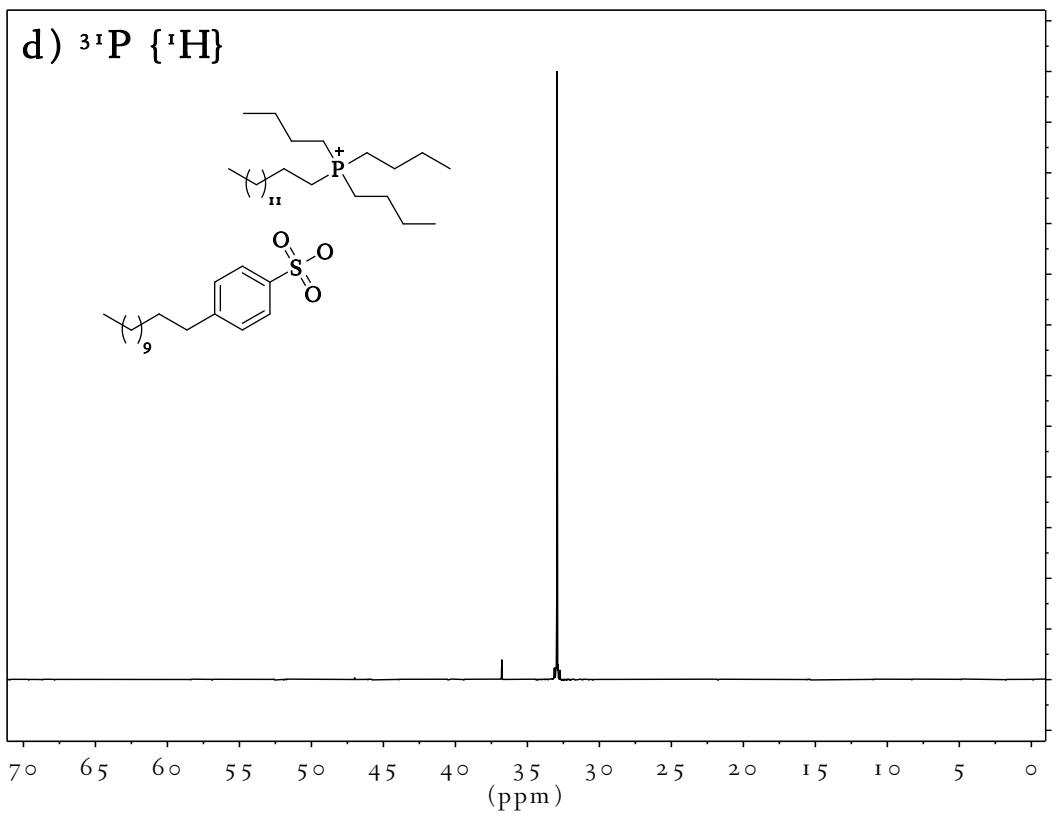

^{1}H NMR (CH_2Cl_2-d_2, TMS, 300MHz) δ$_{H}$/ppm: 7.75-7.67 (m, 2H), 7.20-7.08 (m, 2H), 2.34-2.15 (m, 8H), 1.74-1.41 (m, 18H), 1.40-1.05 (m, 40H), 0.98 (t, 9H, J = 7.5 Hz), 0.93-0.82 (m, 3H). ^{13}C {^{1}H} NMR (CH_2Cl_2-d_2, 75 MHz) δ$_{C}$/ppm: 148.80, 147.25, 146.94, 145.46, 145.41, 127.15, 127.09, 126.39, 125.79, 125.72, 47.64, 45.77, 45.48, 39.73, 39.16, 38.33, 36.96, 36.90, 36.64, 36.51, 31.98, 31.92, 31.89, 31.81, 30.87, 30.67, 29.81, 29.74, 29.70, 29.67, 29.65, 29.63, 29.56, 29.41, 29.35, 29.26, 28.93, 27.72, 27.62, 27.56, 27.27, 24.03, 23.83, 23.66, 23.59, 22.77, 22.68, 22.65, 22.59, 22.13, 21.71, 21.64, 20.62, 19.13, 18.91, 18.50, 18.28, 13.87, 13.84, 13.79, 13.19, 13.18, 11.92. ^{31}P{^{1}H} NMR (CH_2Cl_2-d_2, 121 MHz) δ$_{P}$/ppm: 32.94.

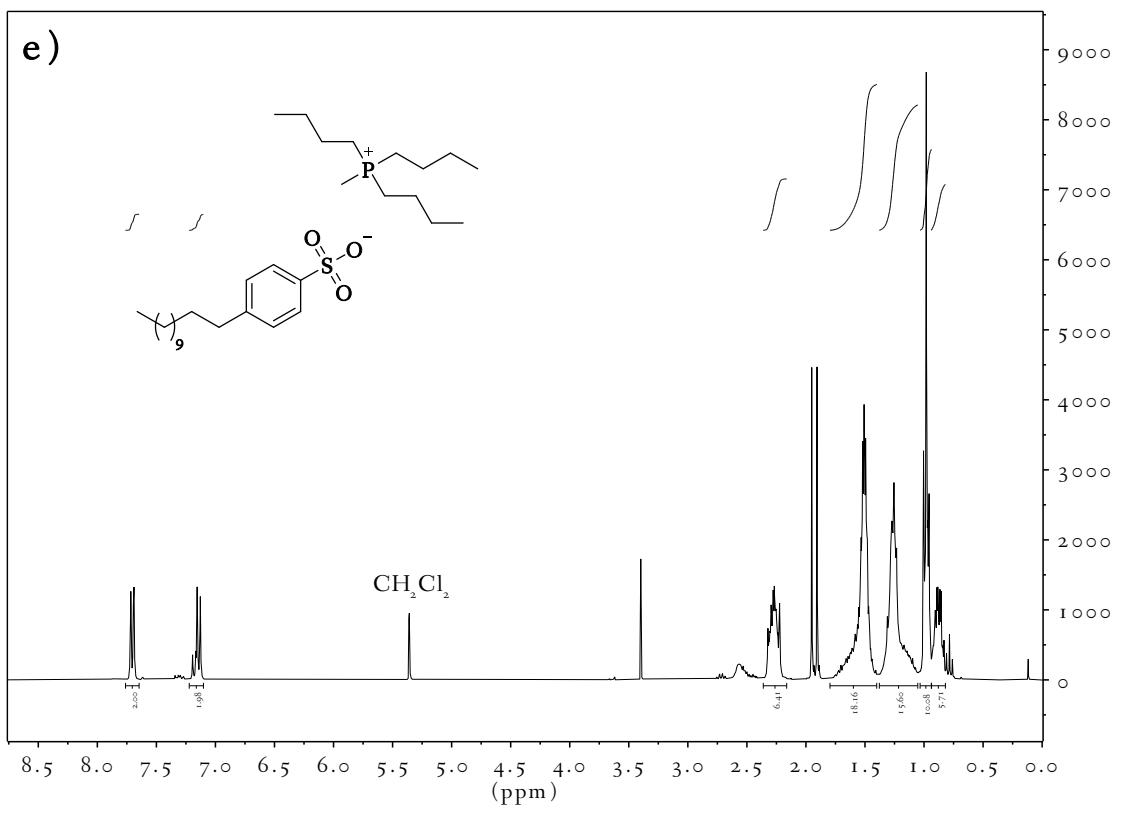

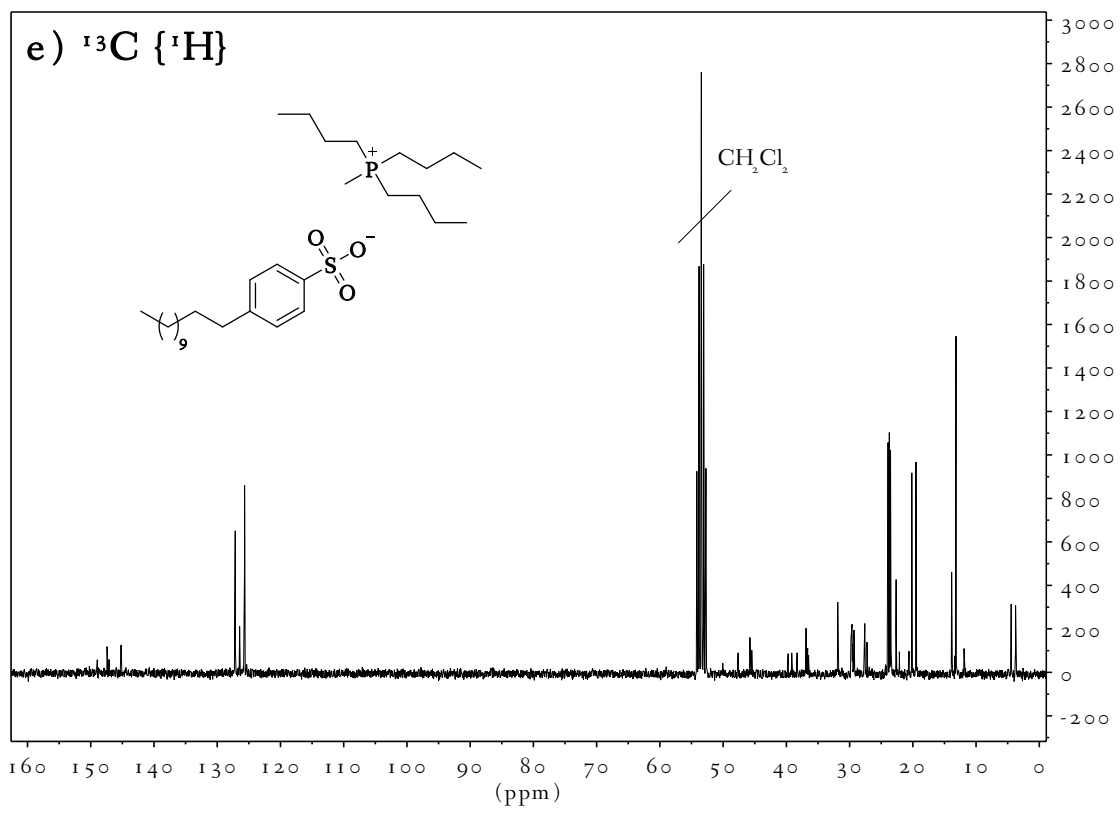

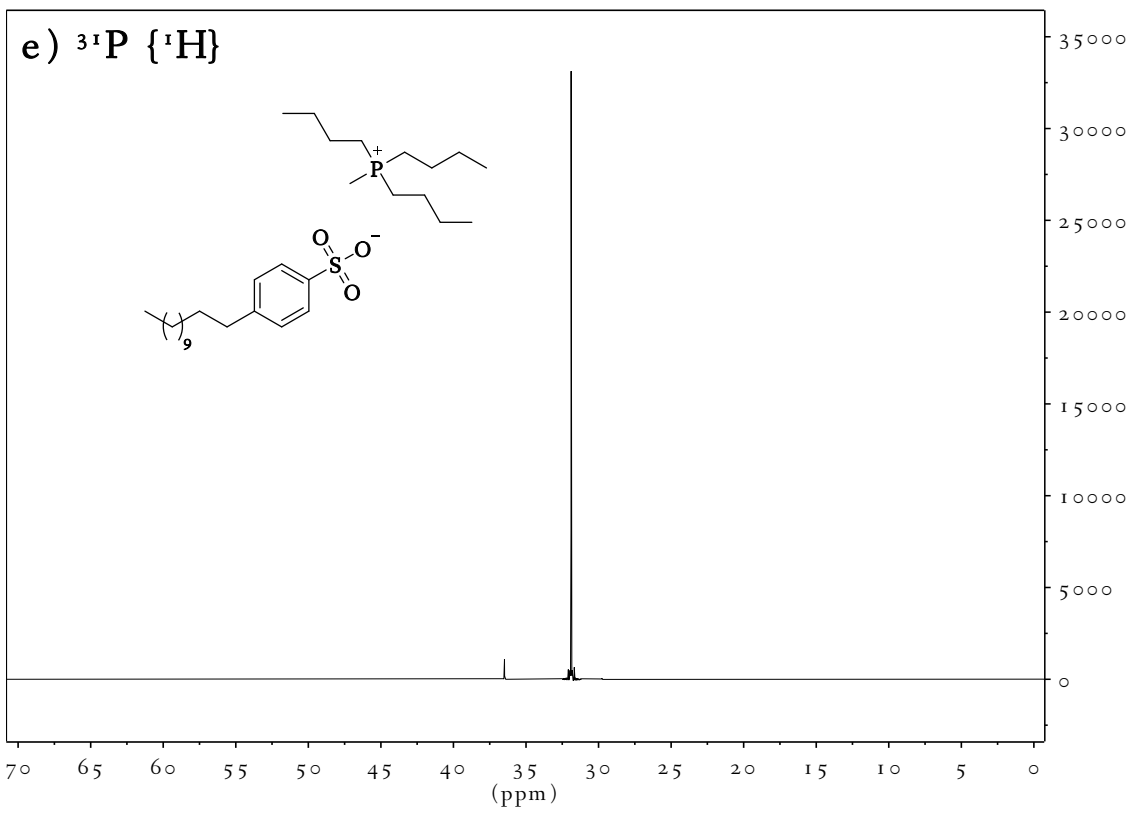

e) ^{1}H NMR (CH_2Cl_2-d_2, TMS, 300MHz) δ_H/ppm: 7.74-7.67 (m, 2H), 7.22-7.10 (m, 2H), 2.37-2.15 (m, 8H), 1.77-1.39 (m, 18H), 1.37-1.06 (m, 20H), 0.98 (t, 9H, J = 6.9 Hz), 0.93-0.82 (m, 3H). ^{13}C {^{1}H} NMR (CH_2Cl_2-d_2, 75 MHz) δ_C/ppm: 148.96, 147.42, 147.11, 145.24, 145.18, 127.22, 127.16, 126.46, 125.74, 125.67, 47.63, 45.76, 45.47, 39.73, 39.15, 38.30, 36.94, 36.88, 36.63, 36.49, 31.96, 31.88, 31.80, 29.79, 29.61, 29.55, 29.39, 29.32, 29.25, 27.70, 27.60, 27.53, 27.25, 23.92, 23.71, 23.53, 23.47, 22.76, 22.67, 22.64, 22.58, 22.12, 20.61, 20.16, 19.51, 13.86, 13.84, 13.78, 13.37, 13.18, 11.91, 4.45, 3.76. ^{31}P{^{1}H} NMR (CH_2Cl_2-d_2, 121 MHz) δ_P/ppm: 31.89.

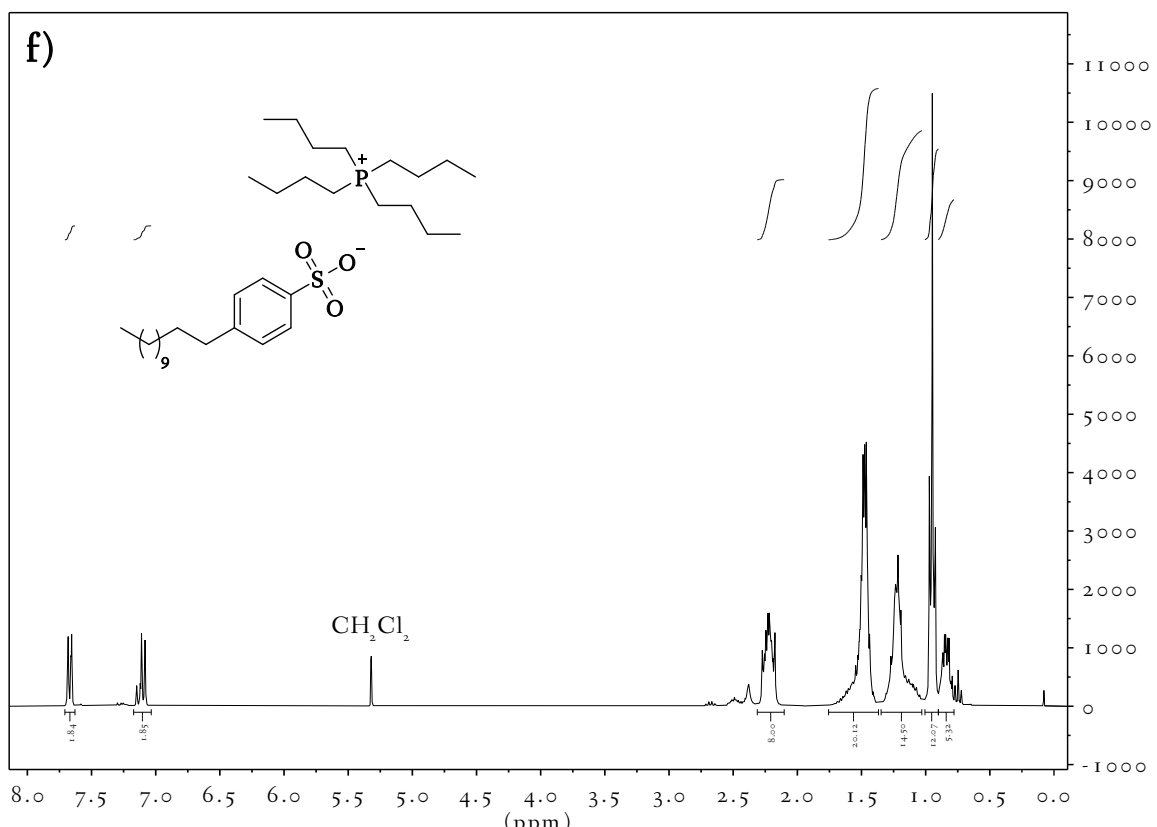

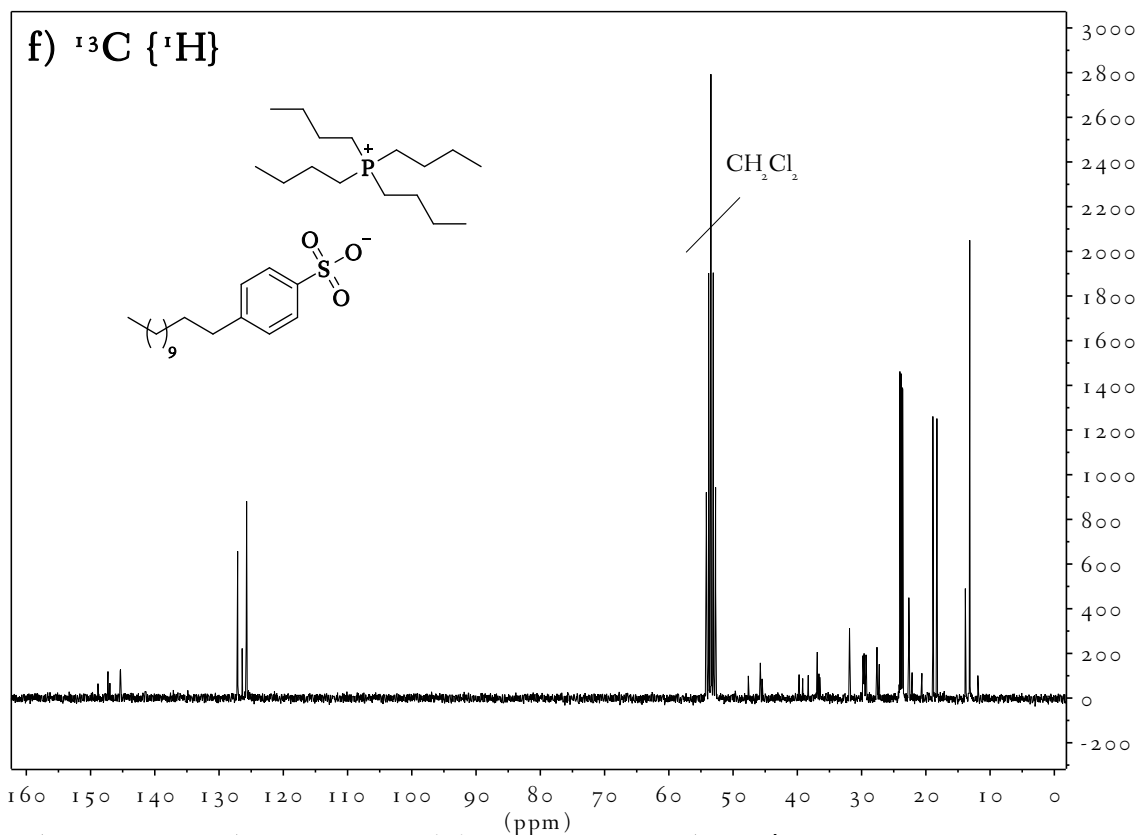

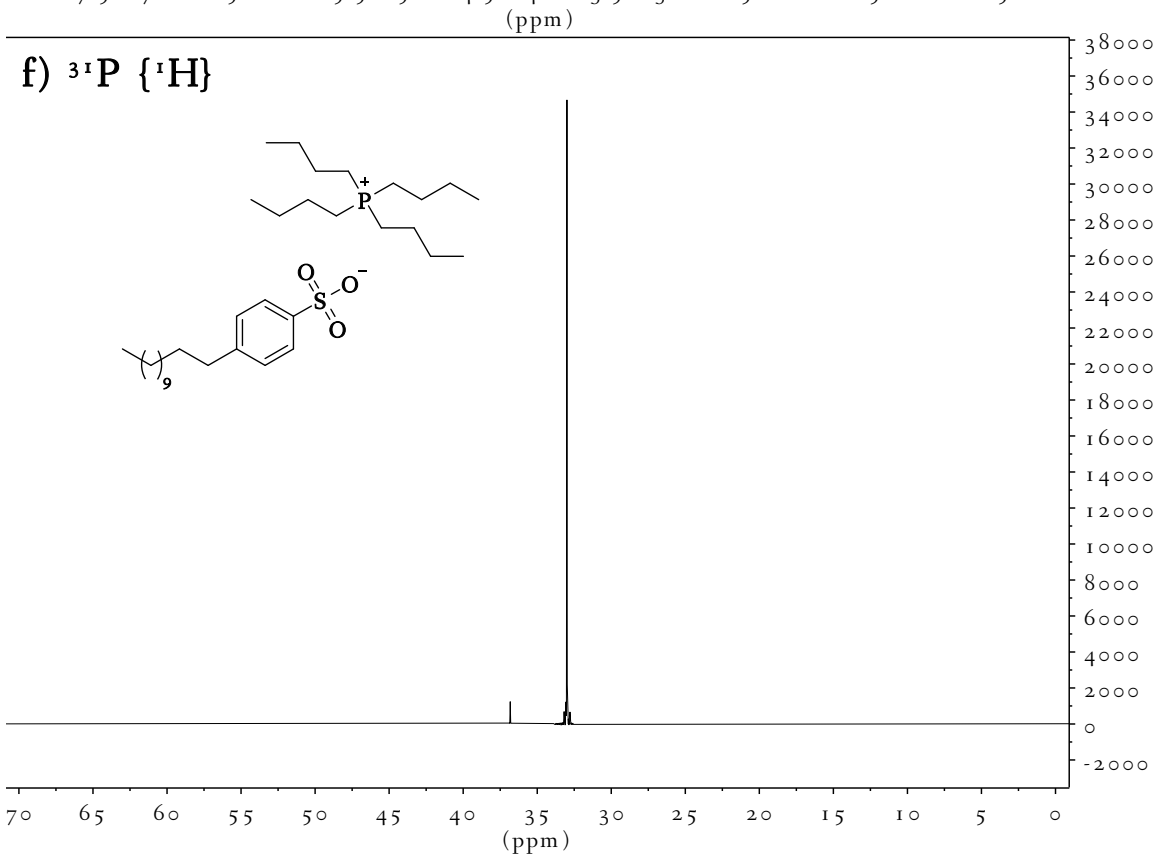

f) ^{1}H NMR (CH_2Cl_2-d_2, TMS, 300MHz) δ_H/ppm: 7.70-7.64 (m, 2H), 7.16-7.05 (m, 2H), 2.30-2.12 (m, 8H), 1.75-1.38 (m, 20H), 1.35-1.03 (m, 18H), 0.95 (t, 12H, J = 6.9 Hz), 0.89-0.79 (m, 3H). ^{13}C {^{1}H} NMR (CH_2Cl_2-d_2, 75 MHz) δ_C/ppm: 148.84, 147.29, 146.98, 145.39, 145.34, 127.17, 127.10, 126.40, 125.78, 125.71, 47.63, 45.76, 45.47, 39.73, 39.16, 38.31, 36.95, 36.90, 36.64, 36.50, 31.97, 31.88, 31.80, 29.79, 29.67, 29.61, 29.55, 29.40, 29.32, 29.25, 27.70, 27.60, 27.54, 27.25, 24.03, 23.83, 23.65, 23.58, 22.76, 22.67, 22.64, 22.59, 22.14, 20.61, 18.91, 18.28, 13.86, 13.84, 13.78, 13.18, 13.17, 11.91. ^{31}P{^{1}H} NMR (CH_2Cl_2-d_2, 121 MHz) δ_P/ppm: 32.99.

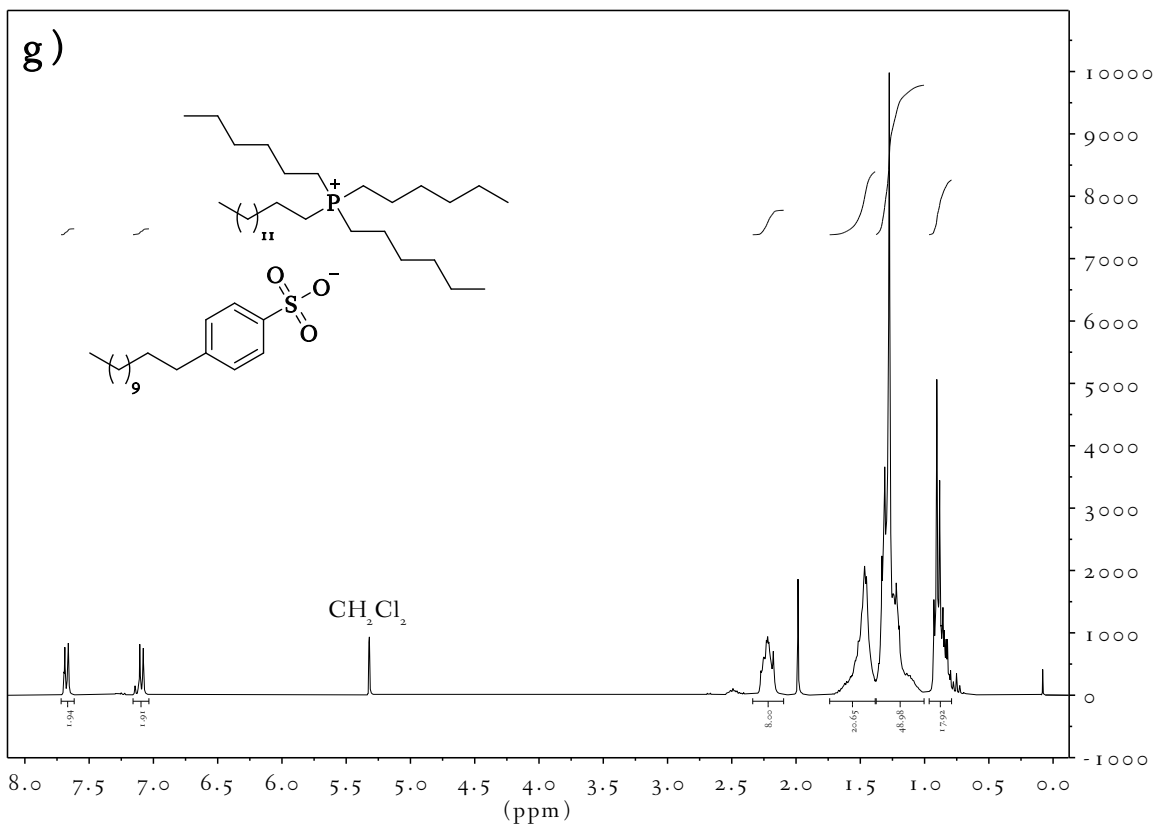

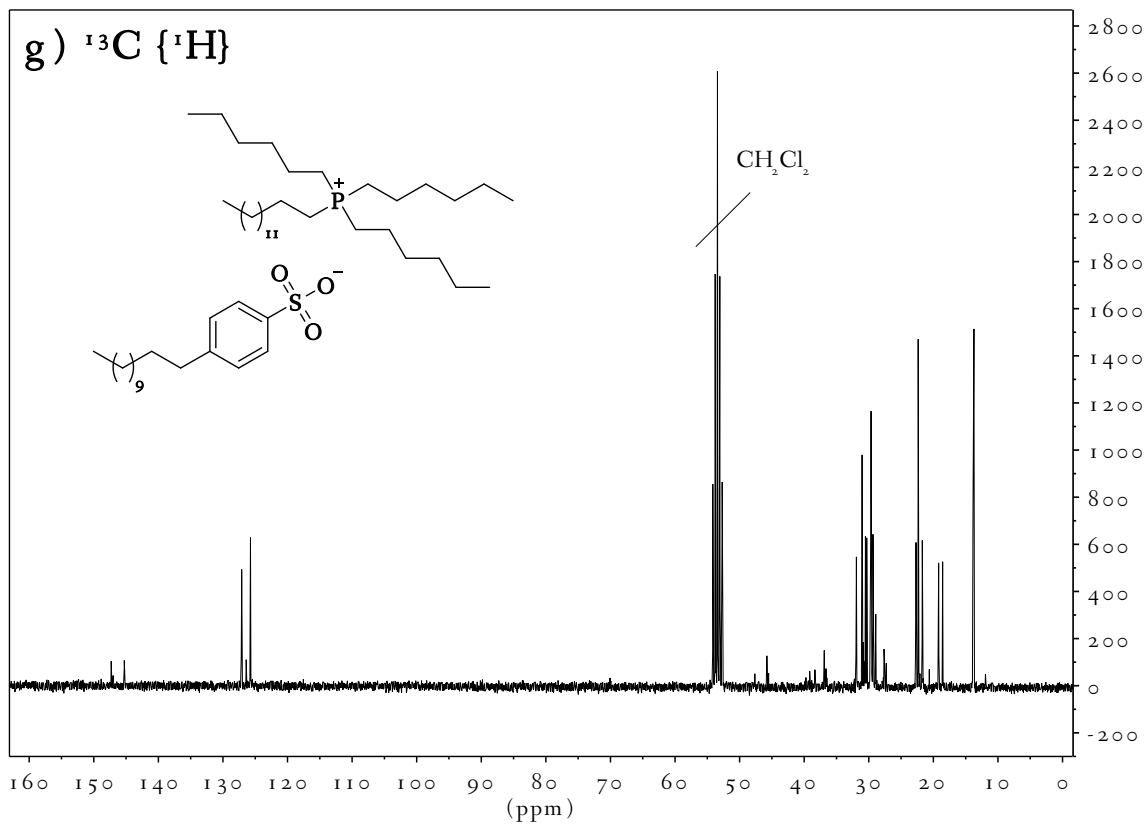

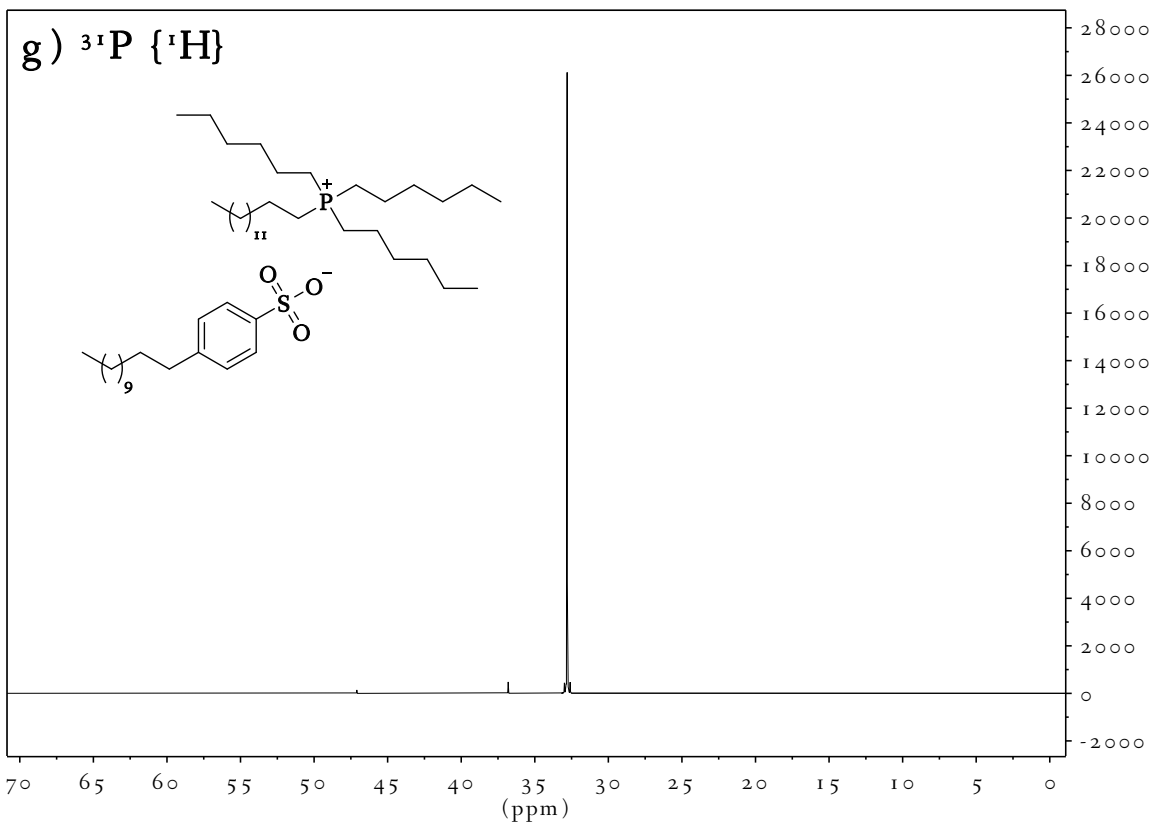

g) **^{1}H NMR (CH_2Cl_2-*d_2*, TMS, 300MHz) δ$_H$/ppm**: 7.73-7.61 (m, 2H), 7.17-7.06 (m, 2H), 2.33-2.13 (m, 8H), 1.72-1.38 (m, 20H), 1.38-1.00 (m, 44H), 0.95-0.81 (m, 15H). **^{13}C {^{1}H} NMR (CH_2Cl_2-*d_2*, 75 MHz) δ$_C$/ppm**: 147.30, 145.27, 127.16, 127.10, 126.40, 125.80, 125.74, 47.65, 45.79, 45.50, 39.15, 38.34, 36.94, 36.89, 36.63, 31.99, 31.92, 31.89, 31.82, 31.04, 30.85, 30.65, 30.52, 30.32, 29.82, 29.70, 29.68, 29.65, 29.56, 29.42, 29.36, 29.33, 28.92, 27.64, 27.57, 27.29, 22.77, 22.68, 22.65, 22.60, 22.35, 21.74, 21.71, 21.67, 21.65, 20.63, 19.18, 18.56, 13.86, 13.84, 13.79, 13.72. ^{31}P{ ^{1}H} NMR(CH_2Cl_2-*d_2*, 121 MHz) δ$_P$/ppm: 32.80.

Figure S2. ^{1}H, ^{13}C{^{1}H} and ^{31}P{^{1}H} NMR spectra of the studied phosphonium-sulfonated ionic liquids: **a**) [$(C_4)_3C_{14}P$][MS], **b**) [$(C_4)_3C_{14}P$][BS], **c**) [$(C_4)_3C_{14}P$][TS], **d**) [$(C_4)_3C_{14}P$][DBS], **e**) [$(C_4)_3C_1P$][DBS], **f**) [$(C_4)_4P$][DBS], **g**) [$(C_6)_3C_{14}P$][DBS].

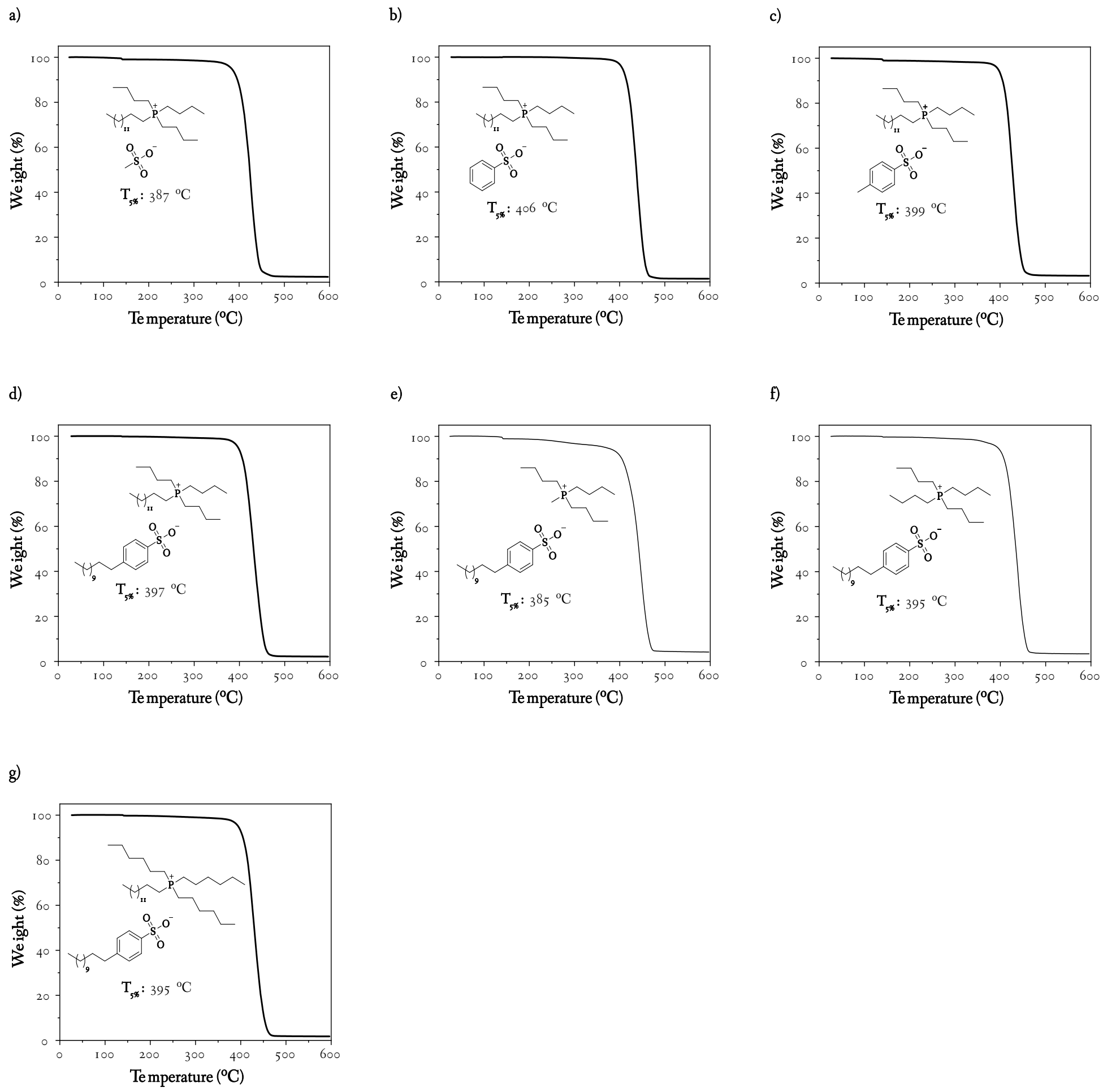

Figure S3. TG profiles of the studied phosphonium-sulfonated ionic liquids: **a**) $[(C_4)_3C_{14}P][MS]$, **b**) $[(C_4)_3C_{14}P][BS]$, **c**) $[(C_4)_3C_{14}P][TS]$, **d**) $[(C_4)_3C_{14}P][DBS]$, **e**) $[(C_4)_3C_1P][DBS]$, **f**) $[(C_4)_4P][DBS]$, **g**) $[(C_6)_3C_{14}P][DBS]$.

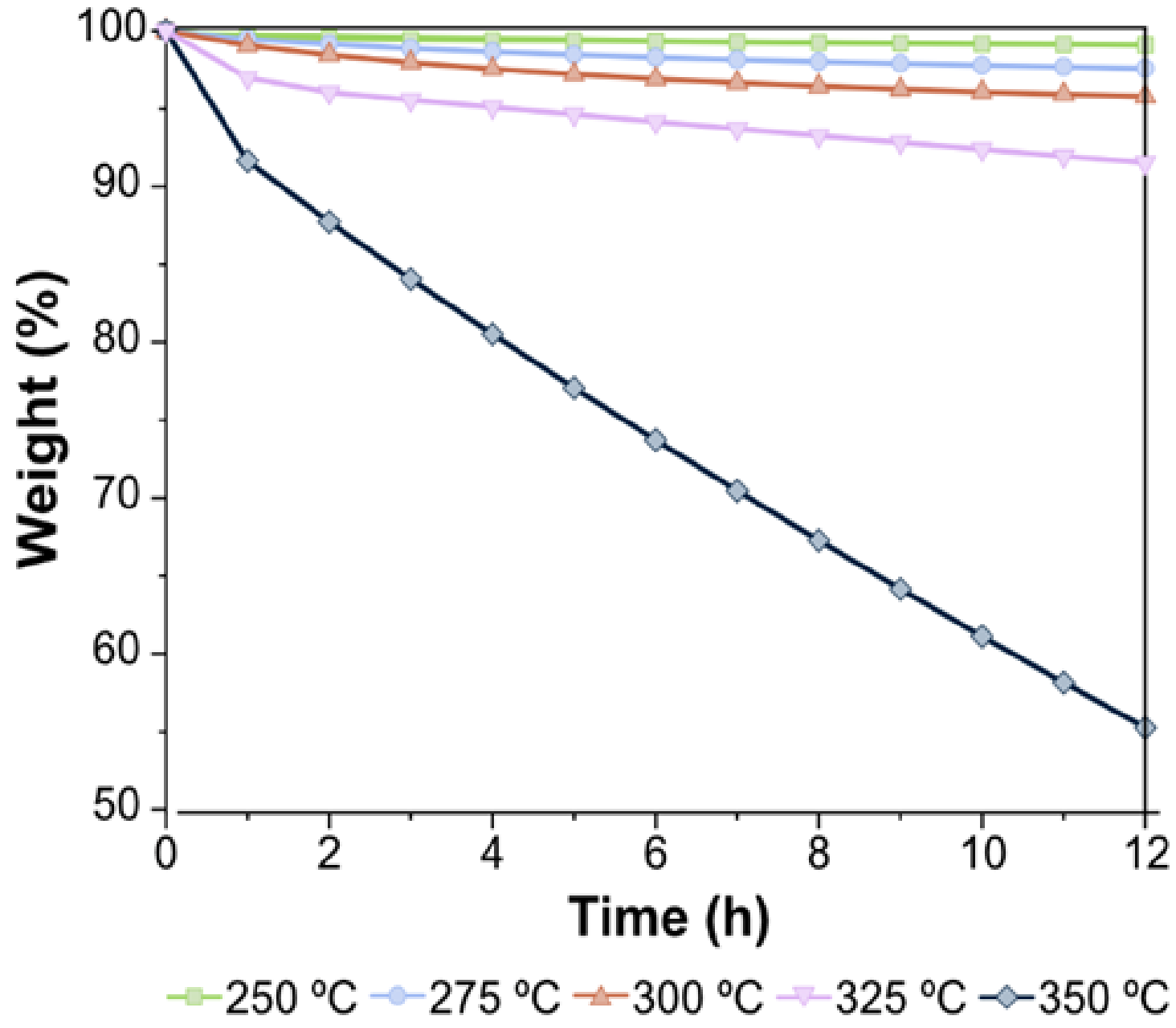

Figure S4. Isothermal TG profiles of [$(C_4)_3C_{14}P$] [DBS] at 250 °C, 275 °C, 300 °C, 325 °C, and 350 °C.

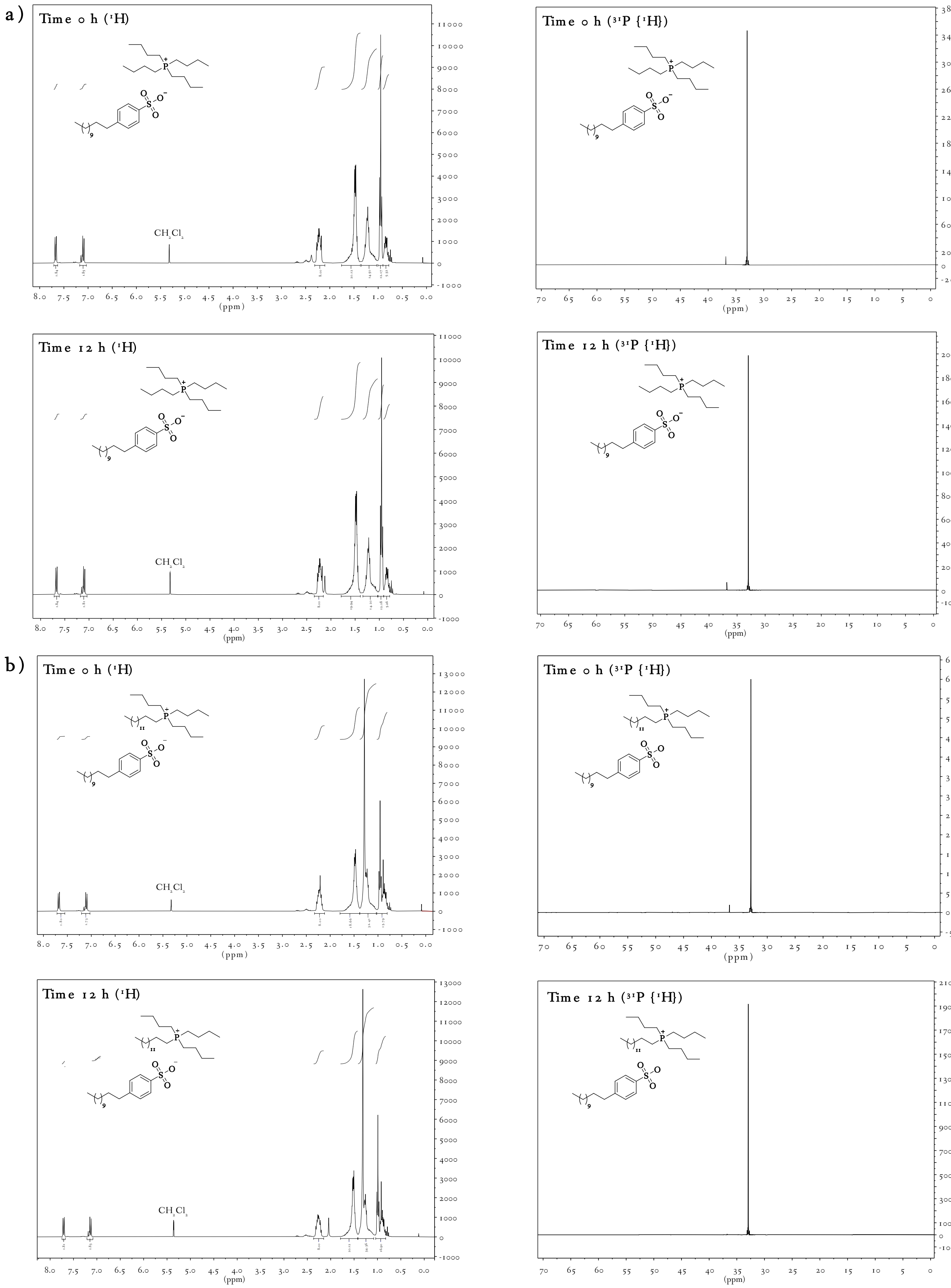

Figure S5. ^{1}H and $^{31}P\{^{1}H\}$ NMR spectra of a) $[(C_4)_4P][DBS]$ and b) $[(C_4)_3C_{14}P][DBS]$ before and after isothermal treatment at 300 °C for 12 h.

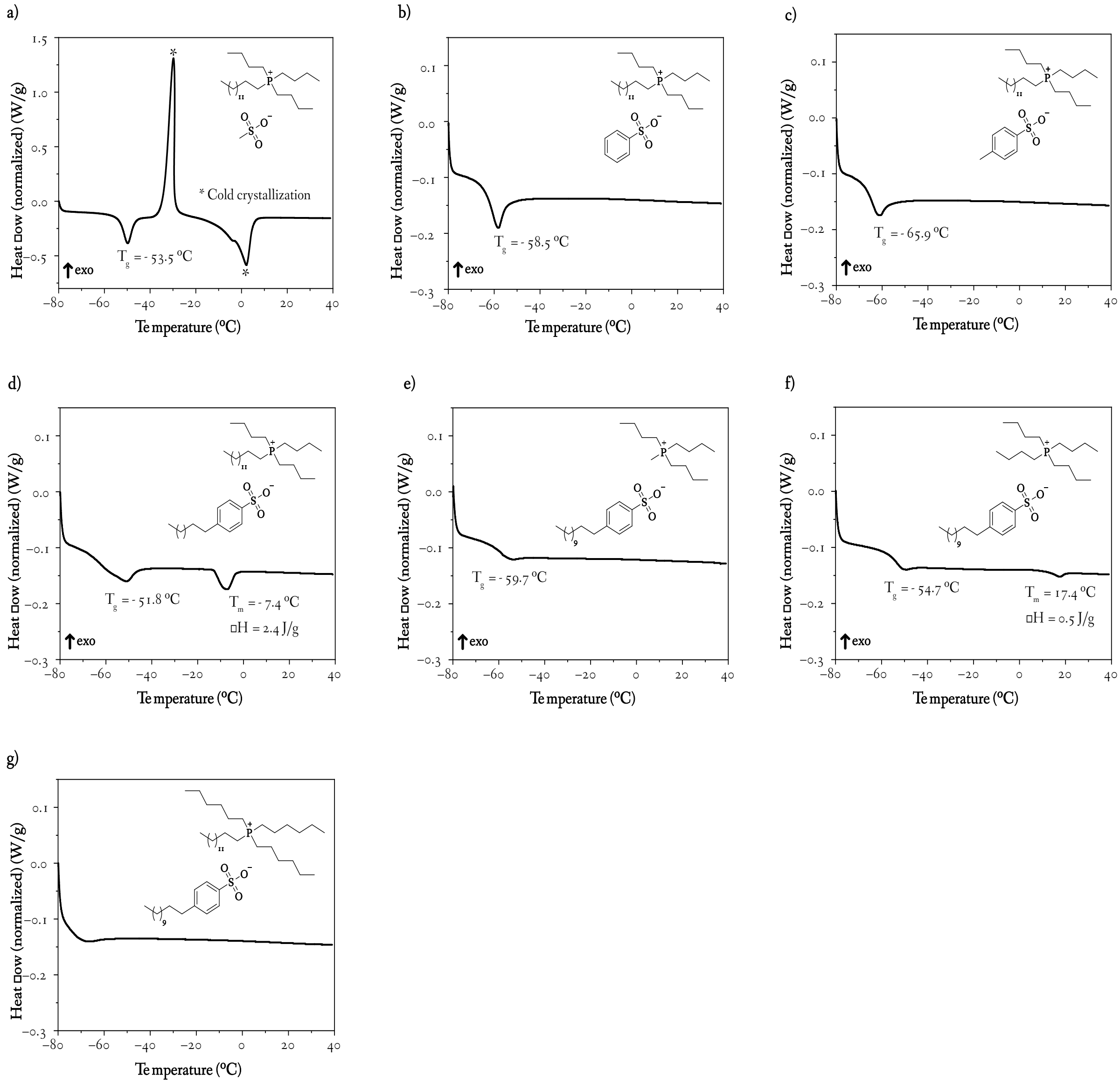

Figure S6: DSC profiles of the studied phosphonium-sulfonated ionic liquids: **a**) $[(C_4)_3C_{14}P][MS]$, **b**) $[(C_4)_3C_{14}P][BS]$, **c**) $[(C_4)_3C_{14}P][TS]$, **d**) $[(C_4)_3C_{14}P][DBS]$, **e**) $[(C_4)_3C_1P][DBS]$, **f**) $[(C_4)_4P][DBS]$, **g**) $[(C_6)_3C_{14}P][DBS]$.

Table S1. Water and chloride content of the studied phosphonium-sulphonate ionic liquids determined by Karl Fischer titration.

IL	Water content (μg/g)	Chloride content (mg/kg)
$[(C_4)_3C_{14}P][MS]$	10076 ± 259	59
$[(C_4)_3C_{14}P][BS]$	2335 ± 204	283
$[(C_4)_3C_{14}P][TS]$	1143 ± 15	547
$[(C_4)_3C_{14}P][DBS]$	1423 ± 10	565
$[(C_4)_3C_1P][DBS]$	13490 ± 297	n.d.
$[(C_4)_4P][DBS]$	3704 ± 152	<10
$[(C_6)_3C_{14}P][DBS]$	1864 ± 114	241

n.d= no determined.

Table S2. Density and linear fitting parameters at 25 °C, 75 °C and 150 °C of the studied phosphonium-sulfonated ionic liquids.

IL	Density, *r* (g.cm^{-3}) 25 °C	75 °C	150 °C	a (g.cm^{-3}.K^{-1})	b (g.cm^{-3})	R^2
$[(C_4)_3C_{14}P][MS]$	0.9520 ± 0.0003	0.9210 ± 0.0004	0.8770 ± 0.0003	5.99×10^{-4}	1.1302	0.9998
$[(C_4)_3C_{14}P][BS]$	0.9800 ± 0.0003	0.9490 ± 0.0003	0.9060 ± 0.0003	5.91×10^{-4}	1.1555	0.9995
$[(C_4)_3C_{14}P][TS]$	0.9760 ± 0.0003	0.9450 ± 0.0004	0.9020 ± 0.0004	5.91×10^{-4}	1.1515	0.9995
$[(C_4)_3C_{14}P][DBS]$	0.9430 ± 0.0000	0.9140 ± 0.0017	0.8700 ± 0.0013	5.84×10^{-4}	1.1173	0.9999
$[(C_4)_3C_1P][DBS]$	0.9790 ± 0.0017	0.9450 ± 0.0007	0.8850 ± 0.0212	7.56×10^{-4}	1.2058	0.9981
$[(C_4)_4P][DBS]$	0.9780 ± 0.0003	0.9470 ± 0.0005	0.9020 ± 0.0009	6.07×10^{-4}	1.1589	0.9999
$[(C_6)_3C_{14}P][DBS]$	0.9320 ± 0.0003	0.9020 ± 0.0003	0.8570 ± 0.0003	6.00×10^{-4}	1.1109	1.0000

Table S3. Viscosity at 25 °C, 50 °C, 75 °C, 100 °C, 150 °C and 200 °C of the studied phosphonium-sulfonated ionic liquids.

Ionic Liquid	Viscosity (□) (mPa·s)					
	25 ºC	50 ºC	75 ºC	100 ºC	150 ºC	200 ºC
$[(C_4)_3C_{14}P]$ [MS]	971.5 ± 62.8	228.8 ± 32.0	73.2 ± 5.1	31.1 ± 1.2	9.8 ± 0.2	4.3 ± 0.1
$[(C_4)_3C_{14}P]$ [BS]	1137.1 ± 54.7	234.0 ± 1.5	74.9 ± 1.6	31.0 ± 0.5	9.2 ± 0.1	4.2 ± 0.0
$[(C_4)_3C_{14}P]$ [TS]	1894.8 ± 77.8	356.7 ± 16.4	101.4 ± 2.7	39.6 ± 0.5	10.7 ± 0.2	4.6 ± 0.1
$[(C_4)_3C_{14}P]$ [DBS]	4685.3 ± 245.6	781.0 ± 8.5	205.6 ± 8.4	74.3 ± 1.5	16.9 ± 0.8	6.5 ± 0.1
$[(C_4)_3C_1P]$ [DBS]	4683.4 ± 715.6	829.7 ± 40.7	219.5 ± 13.4	80.0 ± 2.3	17.8 ± 1.0	6.8 ± 0.4
$[(C_4)_4P]$ [DBS]	6021.2 ± 568.9	890.9 ± 45.7	224.6 ± 5.1	76.0 ± 5.3	17.1 ± 0.1	6.4 ± 0.1
$[(C_6)_3C_{14}P]$ [DBS]	2763.9 ± 120.3	549.6 ± 0.9	164.9 ± 2.2	64.9 ± 1.0	17.6 ± 1.0	9.0 ± 0.1

Table S4. C, H, S, P and O theoretical and experimental values of the studied phosphonium-sulphonate ionic liquids determined by CHNS elemental analysis.

	Theorical				Experimental			
**Ionic liquid**	*C (%)*	*H (%)*	*S (%)*	*P + O (%)*	*C (%)*	*H (%)*	*S (%)*	*P + O (%)*
[$(C_4)_3C_{14}$P][MS]	65.6	11.9	6.5	16.0	64.7	11.8	6.2	17.3
[$(C_4)_3C_{14}$P][BS]	69.1	11.0	5.8	14.1	68.8	11.3	5.5	14.4
[$(C_4)_3C_{14}$P][TS]	69.5	11.0	5.6	13.9	69.0	11.3	5.8	13.9
[$(C_4)_3C_{14}$P][DBS]	72.9	11.7	4.4	11.0	72.8	11.9	4.3	11.0
[$(C_4)_3C_1$P][DBS]	68.7	10.9	5.9	14.5	n.d.	n.d.	n.d.	n.d.
[$(C_4)_4$P][DBS]	69.9	11.1	5.5	13.5	68.9	11.1	6.1	13.9
[$(C_6)_3C_{14}$P][DBS]	74.3	12.0	4.0	9.7	73.9	12.1	3.7	10.3

n.d.= no determined
